# California Autism Prevalence by County and Race/Ethnicity: Declining Trends Among Wealthy Whites

**DOI:** 10.1007/s10803-020-04460-0

**Published:** 2020-03-19

**Authors:** Cynthia Nevison, William Parker

**Affiliations:** 1 Institute for Alpine and Arctic Research, University of Colorado, Campus Box 450, Boulder, 80309-0450 USA; 2 Department of Surgery, Duke University Medical Center, Durham, NC USA

**Keywords:** Autism spectrum disorder, Prevalence, Time trends, California, County, Silicon Valley, Income, Race/ethnicity, Black, White, Hispanic, Asian

## Abstract

County-level ASD prevalence was estimated using an age-resolved snapshot from the California Department of Developmental Services (DDS) for birth years 1993–2013. ASD prevalence increased among all children across birth years 1993–2000 but plateaued or declined thereafter among whites from wealthy counties. In contrast, ASD rates increased continuously across 1993–2013 among whites from lower income counties and Hispanics from all counties. Both white ASD prevalence and rate of change in prevalence were inversely correlated to county income from birth year 2000–2013 but not 1993–2000. These disparate trends within the dataset suggest that wealthy white parents, starting around 2000, may have begun opting out of DDS in favor of private care and/or making changes that effectively lowered their children’s risk of ASD.

**Electronic supplementary material:**

The online version of this article (10.1007/s10803-020-04460-0) contains supplementary material, which is available to authorized users.

## Introduction

Autism spectrum disorder (ASD) is a diagnosis that encompasses a range of severity and can present with co-occurring medical and developmental disorders, including anxiety, epilepsy, attention deficit hyperactivity disorder (ADHD) and intellectual disability (ID) (Rubenstein et al. 2018). ASD is associated with biomarkers that are linked to oxidative stress and inflammation (Goldani et al. 2014; Yang et al. 2015), and is a behavioral condition characterized by impairments in social interaction, communication and restricted or stereotyped behaviors (APA 2013).

The most recent survey of the Autism and Developmental Disabilities Monitoring (ADDM) Network found a mean ASD prevalence of 1 in 59, or nearly 2% of 8 year-olds born in 2006 in selected counties in 11 states (CDC 2018). The 2006 result represented an increase from 1 in 68 in the two previous ADDM surveys in birth years 2002 and 2004 (CDC 2014, 2016). The 2016 National Health Interview Survey (NHIS) estimated a somewhat higher overall prevalence of ASD among American children age 3–17 of 1 in 36, or nearly 3% (Zablotsky et al. 2017). This was up from 1 in 45 in the 2014 NHIS, although the increase was not considered statistically significant (Zablotsky et al. 2017).

ADDM is a biannual surveillance system conducted in selected regions of the United States that focuses on 8 year-old children. ASD cases are determined by systematic review and abstraction of information from existing health and education-based evaluations, followed by independent scoring and analysis by experienced clinicians. NHIS is a nationally representative survey of the U.S. population conducted as an in-person and/or telephone interview, in which a sample child in the household between 3 and 17 years old is selected and parents are asked, “Has a doctor or health professional ever told you that your child had Autism, Asperger’s disorder, pervasive developmental disorder, or ASD?” In general, the NHIS survey collects less detailed information than ADDM about ASD cases but includes a wider age cohort range.

Historically, diagnosed ASD prevalence was 1 in 150 in the ADDM survey of 8 year-olds born in 1992, 1 in 2500 in the early 1980s, and as low as 1 in 100,000 in the 1930s (McDonald and Paul 2010; CDC 2007; Nevison et al 2018). Thus, diagnosed ASD prevalence has increased significantly over time in the United States. However, several recent studies have found slowing or plateauing rates of growth in ASD (Zablotsky et al. 2017; CDC 2018; Pearl et al. 2019; Nevison and Zahorodny 2019). Further, when the time trends have been resolved by race/ethnicity, the slowdown appears to be occurring mainly among white children, with ongoing increases occurring among children of other races. Indeed, CDC (2018) attributed the 15% increase in ASD between birth year 2006 and the two previous ADDM reports largely to the narrowing gap between white children and black and Hispanic children, who historically had lower rates of diagnosed ASD prevalence.

Nevison and Zahorodny (2019) examined a more recent Department of Education dataset extending through birth year 2013 and concurred that ASD rates were increasing faster among black and Hispanic children than among whites. However, they found that the rate of ASD among blacks had not merely caught up to but had actually surpassed the rate among whites in the majority of states. Furthermore, while autism prevalence among whites plateaued between birth years 2003–2007, it resumed its increase after birth year 2007 and increased among all races nationwide between birth years 2007 and 2013.

Among the recent studies identifying a slowdown or plateau in ASD rates, to date only one has reported on the socioeconomic profiles of the ASD cases (Pearl et al. 2019). That study used health insurance as a proxy for income level and noted, strikingly, that ASD prevalence had reached a near plateau among privately-insured (i.e., wealthier) white children, but had continued to increase among publicly-insured whites.

This paper examines a late 2019 age-resolved snapshot of ASD data from the California Department of Developmental Services (DDS) that is broken down by race/ethnicity as well as by county. DDS historically has served the more severe end of the autism spectrum (California DDS 1999, 2002; Nevison et al. 2018). The DDS dataset permits the estimation of race-specific time trends in ASD prevalence among white, Hispanic, black and Asian children across 36 California counties, encompassing a range of income levels. The principal goal is to understand the apparent recent plateau in ASD prevalence and to evaluate whether it is occurring preferentially among specific income and/or race/ethnicity groups.

## Methods

### ASD Counts from DDS

The current study uses California Department of Developmental Services (DDS) autism counts distinguished by county, by birth year from 1993 to 2013, and by race/ethnicity for each of these groups: white, Hispanic, black and Asian (including Hawaiian and Pacific Islanders) and All Races. Since data were de-identified (i.e., blanked out) for all cells with < 11 consumers, only the 36 most populous California counties were considered (out of 58 total). DDS provided additional race-resolved ASD counts statewide (including all 58 counties) and for 10 selected groups of 2 or more adjacent counties (within the 36 most populous), in which the aggregation of data across counties pushed the totals to 11 or above. For several counties, including Marin, Imperial, Madera, Merced and San Luis Obispo, it was possible to calculate the county specific population for Hispanics and/or whites by subtracting the counts for all the other individual member(s) from the county group total. Since the relevant data did not include identifying information and since the datasets were aggregated by age at the county level, this study did not require institutional review and approval.

The autism counts were obtained through a direct request to DDS and reflect a snapshot of the DDS Code 1 autism caseload as of late October 2019. While the definition of Code 1 has changed several times over the years within the DDS system (California DDS 2019), by October 2019, the entire caseload had been switched over to the DSM-5 definition of autism spectrum disorder (ASD). However, the cases entering the system prior to 2014 may have been diagnosed originally using the DSM-IV nomenclature, which distinguished full syndrome autistic disorder (AD) from milder forms of autism including Asperger’s syndrome and PDD-NOS (APA 1994).

DDS provides services to eligible individuals living in California who meet the DSM-5 diagnostic criteria (APA 2013) for autism spectrum disorder (ASD). To qualify for services, these individuals also must demonstrate significant functional disability in three out of seven life challenges, which include self-care, language, learning, mobility, self-direction, capacity for independent living and economic self-sufficiency. DDS services for the young (age 6–26 years) population considered in our dataset include community care facilities, day care programs, and in-home and out-of-home respite care. However, the bulk of spending is grouped under the more general categories of “Support Services” and “Miscellaneous,” which include over 18 and 100 separate categories, respectively, such as behavior management consultations and public school early intervention programs. Notably, medical costs are borne by families and private or public health insurance programs and are not typically part of DDS services (Leigh et al. 2016).

### NCES Total School Populations (Denominators for Prevalence Calculation)

Autism prevalence was computed by dividing the DDS ASD counts by total public school populations, as provided by the National Center for Education Statistics (NCES) (https://nces.ed.gov/ccd/elsi/). The data were obtained in annual reports, compiled in fall, for school years extending from 1998–99 to 2012–2013 biannually and 2013–14 to 2017–18 annually. (1998–99 was the first year that data became available partitioned by both grade and race.) Each dataset was broken down by California county, race and by grade from kindergarten to 12th grade. Total and race/ethnicity-specific populations were obtained for each county and grade. Up until 2007–08, the NCES data were partitioned into 5 race/ethnicity groups, including American Indian or Alaska Native (AIAN), white, Hispanic, black, and a single group encompassing all Asians. From 2008 to 2009 onward, NCES reports Native Hawaiian or Other Pacific Islanders separately from Asians. However, these were combined into a single group encompassing all Asians for the ASD prevalence calculations.

Since the DDS ASD numerators were from Fall 2019, while the most recently available NCES denominators were from Fall 2017, the NCES populations were extrapolated to report year 2019 using linear regressions of population(iry, iby) v. NCES report year, where iby = birth year (1993–2013) and iry = report year (1998–2017). In these calculations, birth year was estimated as a function of school grade (used as a proxy for age) and report year: iby = iry − iage, where iage ranged from 6 years old (1st grade) to 17 years old (12th grade). The kindergarten populations (age 5) were not used in the linear extrapolations because they tended to be slightly higher than the subsequent grades for any given birth cohort in many counties.

The extrapolation approach yielded county and race-specific populations that were generally stable or smoothly linear for each California county (Supplementary Figs. S1 and S2), lending confidence that the projected 2019 populations were reasonable estimates of the true populations. However, the approach involves some uncertainty and the most recent birth years (> 2008) are covered by only a few NCES reports. For example, birth year 2009 is covered by only three NCES reports, reflecting children who were 6, 7 and 8 years old in the 2015–16, 2016–17 and 2017–18 school years, respectively, yielding only 3 data points for the linear extrapolation to 2019. The 2019 populations of the 2009–2013 birth cohorts therefore were assumed to equal the 2008 birth year extrapolation.

## Results

### Statewide California ASD Prevalence Trends by Race/Ethnicity

**Fig. 1:**
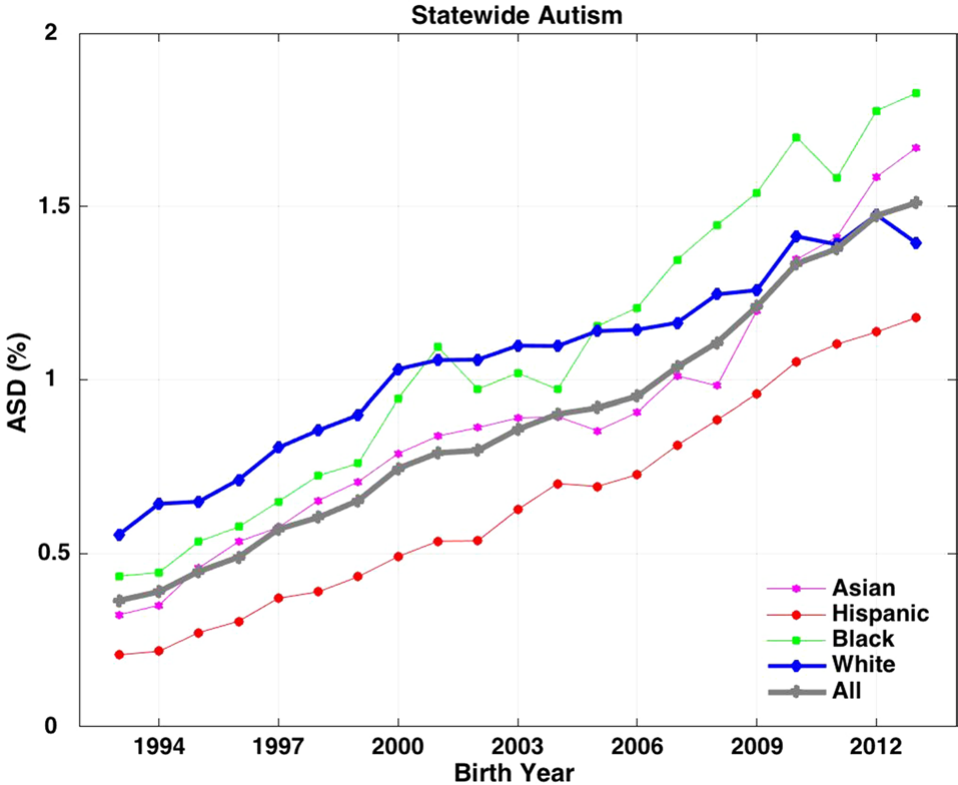
Statewide California ASD prevalence among race/ethnicity groups based on an age-resolved snapshot of ASD data from Fall 2019

ASD prevalence statewide (summing over all 58 California counties) reached 1.5% by birth year 2013 in the DDS dataset (Fig. 1). Highest prevalence occurred among black children (1.8%), followed by Asian (1.7%), white (1.4%) and Hispanic children (1.2%). While ASD prevalence among all race/ethnicity groups increased more or less continuously from birth year 1993–2013, a discernible flattening in the growth trend occurred around birth year 2000 for all groups except Hispanics and was particularly evident among whites. The flattening persisted only a few years for blacks and somewhat longer for whites and Asians, but by the mid to late 2000s all groups had resumed some degree of upward growth in ASD prevalence.

### Autism Prevalence Trends by Race/Ethnicity and County

**Fig. 2:**
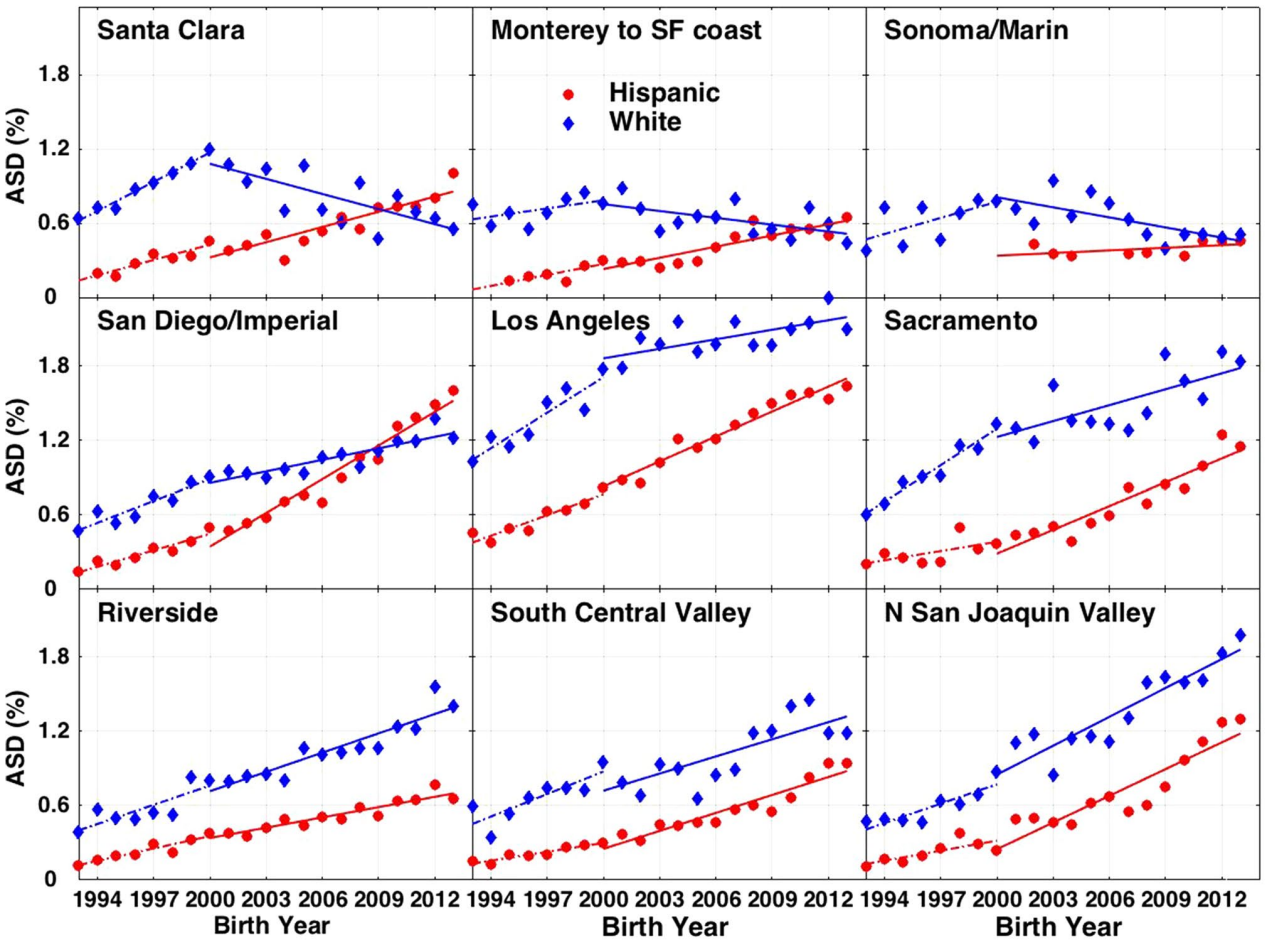
ASD prevalence vs. birth year for Hispanics (red circles) and whites (blue diamonds) in 9 selected California counties or county groups. Linear regressions distinguish time trends for two separate periods: 1993–2000 (dashed) and 2000–2013 (solid). “Monterey to SF Coast” includes Monterey, Santa Cruz, San Mateo and San Francisco Counties. “South Central Valley” includes Fresno, Kern, Kings, Madera and Tulare Counties. “North San Joaquin Valley” includes Merced, San Joaquin and Stanislaus Counties. Top row shows higher income counties; middle row are middle income counties; bottom row shows lower income counties

The ASD prevalence vs. birth year trend curves evolve in notably different ways among counties and between whites and Hispanics (Fig. 2; see also Supplementary Fig. S3 for the complete set of California counties and county groups). Among whites, ASD prevalence had a positive growth trend between birth years 1993–2000 in all counties. Thereafter, the trends diverged. In the wealthiest counties, including Santa Clara and the coastal counties extending from Monterey through San Francisco and north to Marin and Sonoma, white ASD prevalence decreased over birth year 2000–2013 (Fig. 2, top row). In contrast, in middle income counties, including the large metropolitan areas of San Diego, Los Angeles and Sacramento, ASD prevalence continued to increase across birth year 2000–2013, but at a slower rate than across birth year 1993–2000 (Fig. 2, middle row). Finally, in lower income counties, spanning urban, agricultural and rural areas of California, ASD prevalence increased at a comparable or accelerating rate over birth years 2000–2013 compared to birth years 1993–2000 (Fig. 2, bottom row).

Hispanics are the largest race/ethnicity group in California, accounting for half or more of the total public school population over the time frame of this study (Supplementary Fig. S2). In contrast to whites, ASD prevalence among Hispanics increased continuously over birth years 1993–2013 in almost all California counties, with the increases occurring at a comparable or accelerated rate from 2000 to 2013 compared to 1993–2000. Conversely, Hispanic prevalence was substantially lower than white prevalence in most counties, especially at the beginning of the DDS dataset in birth year 1993. However, the differential growth rates result in a crossover in some counties, including San Diego, Santa Clara and the wealthy coastal counties extending from Monterey to Sonoma, where Hispanic ASD prevalence exceeds white prevalence at the end of the DDS dataset in birth year 2013. Moreover, in Orange County and the East Bay counties of Alameda and Contra Costa, the differential growth rates result in comparable prevalence levels among Hispanics and whites by birth year 2013 (Supplementary Fig. S3).

Figure 2 focuses on whites and Hispanics, since they are the dominant race/ethnicity groups across the large majority of California counties (Supplementary Fig. S1). However, ASD trends for blacks and Asians also are shown in Supplementary Figs. S4 and S5, respectively, with each compared to white ASD trends. Blacks are a minority in California and sufficient data to estimate prevalence were available only in 9 counties or county groups. In these, black ASD prevalence exceeded white prevalence in 5 counties, was comparable in Riverside and the San Joaquin Valley counties, and was slightly lower than white prevalence in Los Angeles. Black prevalence increased in all available counties over birth years 2000–2013, at a steeper rate than white prevalence in seven counties and at a comparable rate in the San Joaquin Valley (Supplementary Fig. S4).

Asians comprise the third largest segment of the California school population, after Hispanics and whites (Fig. S2). Sufficient data to estimate Asian ASD prevalence trends were available in 17 counties or county groups (Supplementary Fig. S5). Asian ASD trends in the wealthy counties of the San Francisco Bay Area show some of the same tendencies as white ASD trends, with a steep rise over birth years 1993–2000 followed by flatter trends over birth years 2000–2013. Unlike white ASD prevalence, Asian ASD prevalence in Santa Clara and the coastal counties of Monterey-to-San Francisco merely flattened over 2000–2013 rather than actually declining. In most counties, Asian ASD prevalence started out lower than or comparable to white prevalence in birth year 1993 but ended up comparable to or higher than white prevalence by birth year 2013.

**Fig. 3:**
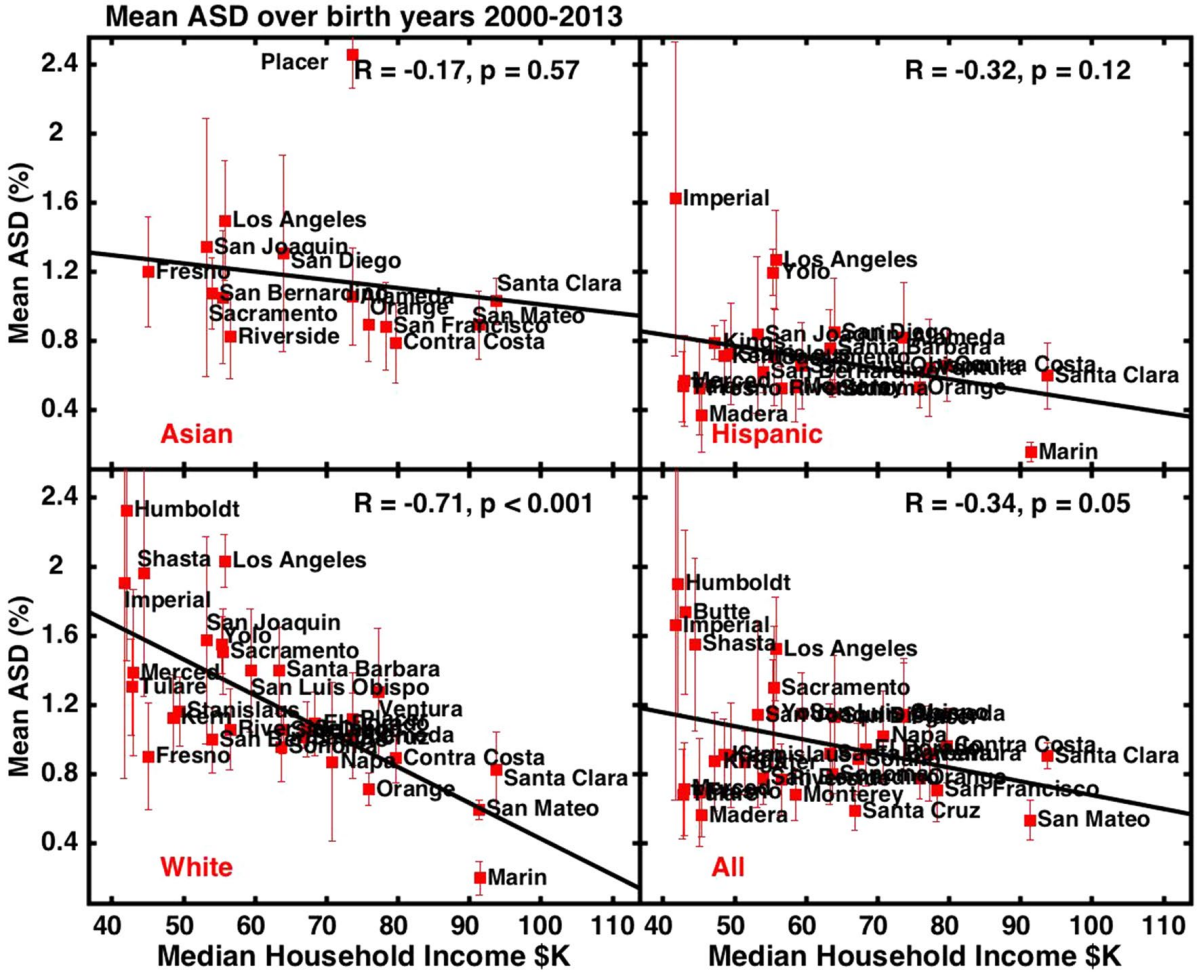
Mean ASD prevalence over birth years 2000–2013 by California county. Error bars show the standard deviation of the mean. Results are shown for four different race/ethnicity groups

At the county level, mean ASD prevalence over birth year 2000–2013 is significantly and inversely correlated to median household income (US Census Bureau 2014) for whites (R =  − 0.71, p < 0.001) but is not correlated for Asians, Hispanics, or blacks (Fig. 3). For all races combined, a weak inverse correlation persists (R =  − 0.34, p = 0.05) due largely to the strength of the relationship among whites. Over the earlier period of the data, birth year 1993–2000, mean county-level ASD prevalence is completely uncorrelated to income for any race (Supplementary Fig. S6).

**Fig. 4:**
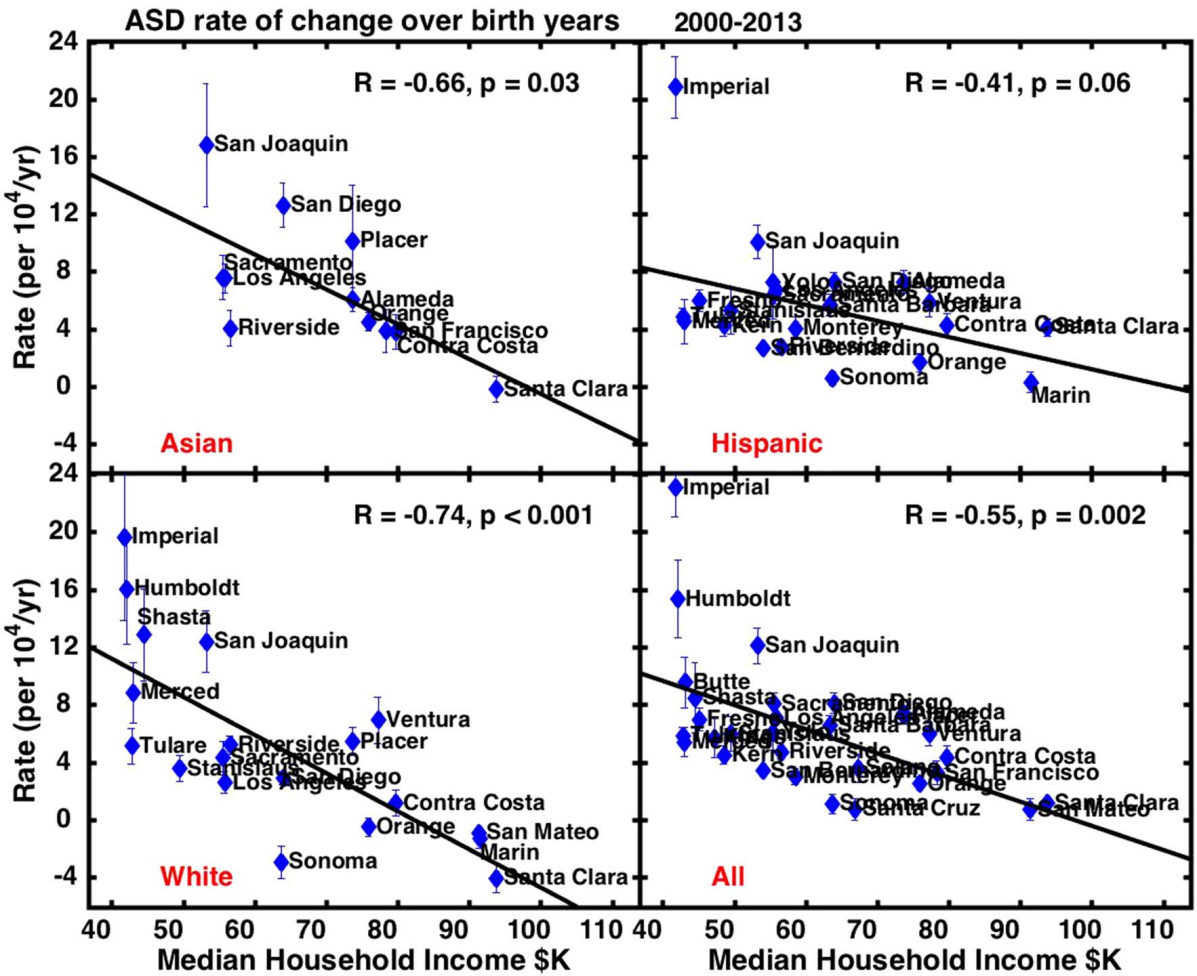
Rate of change in ASD prevalence over birth years 2000–2013 by California county, estimated using linear regression. Error bars show the error in the linear regression slope. Counties are included only when the slope error is < 40% of the slope. Results are shown for 4 different race/ethnicity groups

The rate of change in county-level ASD prevalence over birth year 2000–2013 is also significantly and inversely correlated to median household income for whites (R =  − 0.74, p < 0.001), all races (R =  − 0.55, p = 0.002) and also for Asians (R =  − 0.66, p = 0.03), but not for Hispanics (Fig. 4) or blacks. In contrast, the rate of change over birth year 1993–2000 is uncorrelated to income at the county level for any race (Supplementary Fig. S7).

## Discussion

ASD prevalence among blacks statewide in California, at 1.8% in birth year 2013, was higher than any among other race/ethnicity group statewide in the DDS dataset (Fig. 1). This finding is consistent with Nevison and Zahorodny (2019), who showed that black ASD prevalence in Individuals with Disabilities Education Act (IDEA) data, while historically lower than white prevalence, has not only caught up to white prevalence in recent years but substantially surpassed it in the majority of states. These observations challenge the theory that ASD prevalence is largely a function of awareness and access to services (Liu et al. 2010; Mazumdar et al. 2010), since black ASD rates would be expected to match, but not exceed, white rates once they attained the same level of care.

Historically, black and Hispanic children have had lower access to ASD evaluation and care than white children and also have been diagnosed on average at a later age (Mandell et al. 2002; Liptak et al. 2008). More recently this gap has narrowed, thanks in part to the State Children’s Health Insurance Program (CHIP), which has expanded insurance coverage for lower income families, especially Hispanic and African American populations (Liptak et al. 2008; Kaiser Family Foundation 2019). The most recent studies have reported mixed results with respect to lingering access-to-care disparities. Some have found few obvious differences among whites and non-whites in health care-based screening rates, referral practices and final diagnosis following evaluation by pediatricians (Augustyn et al. 2019; Bilaver and Havlicek 2019), while others have found that non-whites are still less likely than whites to have an official clinical diagnosis despite having identifiable markers of ASD in their health and/or education records (Wiggins et al. 2019).

A number of the studies evaluating racial disparities in access to care have combined blacks and Hispanics into a single “non-white” category, but recent trend analyses suggest that ASD rates among these groups are evolving in different ways (CDC 2018; Pearl et al. 2019; Nevison and Zahorodny 2019). The statewide DDS data in Fig. 1 are consistent with the suggestion that access-to-care issues may affect blacks and Hispanics differently. In contrast to black prevalence, ASD prevalence statewide among Hispanics still lags well behind whites (at 1.2% and 1.5%, respectively, among 6 year-olds born in 2013). This finding contrasts with the results of Nevison and Zahorodny (2019), whose study of 3–5 year-old ASD prevalence in California IDEA data found that Hispanic and white children had a comparable ASD prevalence of 1.2% by birth year 2013. The disparate DDS and IDEA results could reflect ongoing hesitance among recent immigrants to apply to state-funded agencies like DDS for aid, contrasted with a greater level of comfort using services from their local school districts, which are guaranteed by IDEA (California DDS 2001; Fountain and Bearman 2011; USDE 2019).

The county level DDS data unmask patterns that are hidden in aggregated statewide data. Most notably, the decelerated but still ongoing increase in statewide white ASD prevalence after birth year 2000 (Fig. 1) is the net result of strikingly different trends at the county level, involving steep increases in lower income counties contrasted with declines in high income counties (Fig. 2). Birth year 2000 is a change point in the county-level data between an earlier period, 1993–2000, when white ASD prevalence increased at a similar rate in most counties, and a later period, 2000–2013, when white ASD trends diverged sharply across county income lines. The early period began just a few years after the onset of the rapid rise in diagnosed ASD, whose starting point has been tagged to about birth year 1988 (McDonald and Paul 2010). The later period marks a time of greater awareness, information, and growing concern about rising rates of ASD, particularly in California (California DDS 1999, 2003).

During the early period, 1993–2000, the DDS data show no correlation with wealth at the California county level, for any race/ethnicity group, in either absolute ASD prevalence or the rate of growth in prevalence (Supplementary Figs. S6, S7). Furthermore, white ASD prevalence at that time was generally higher than other race/ethnicity groups, and the white ASD growth rate was positive in all counties. Then, starting at approximately birth year 2000, wealthy California counties saw a flattening or an actual decline in white ASD prevalence, while lower income counties experienced a continuous increase or even an acceleration in the rate of increase (Fig. 2). ASD trends among California Asians (but not blacks or Hispanics) display some of these same patterns, albeit more weakly, as discussed further below.

By birth year 2013, the absolute ASD prevalence among whites in the lowest income counties was at least double that of whites in the highest income counties and as much as a factor of 10 higher (comparing Humboldt and Marin Counties). These results reflect a dramatic departure from the traditional perception that ASD is a diagnosis found predominantly among whites of high socioeconomic status (Schopler et al. 1979; Durkin et al. 2017). They also contradict the view that ASD prevalence patterns and recent trends can be explained by diagnosis increasing rapidly among communities of color while remaining static across white populations. Here it is pertinent to note the well-established relationship between education levels and income. Greater wealth leads to higher educational attainment (White 2015) while higher educational attainment leads to higher earnings (Torpey 2018).

The ASD trends among whites and Asians in Santa Clara County, home of the Silicon Valley, are particularly intriguing with respect to the role of wealth and education. In the 1990s, Santa Clara County experience a rapid increase in the rate of ASD, with a doubling in prevalence among whites and Asians in just seven years from 1993 to 2000. The absolute ASD prevalence among whites in 2000 (1.2%), was among the highest in all California counties (Figs. 3 and S6). These high rates helped give rise to influential theories about “assortative mating” and the role of “folk physics” vs. “folk psychology,” in which men with poor social skills but strong ability and education in math and engineering were able to find mates and thereby father genetically autistic children (Baron-Cohen et al. 1997; Baron-Cohen 2006). These theories are contradicted by the decline or flattening of ASD rates among the white and Asian populations of the Silicon Valley. Notably, by the latter half of the DDS record, Santa Clara white ASD prevalence was among the lowest of all California counties (Figs. 3, S6).

Rather than supporting genetic explanations, the DDS data tend to implicate environmental factors as underlying the etiology of ASD. For example, the lower prevalence of ASD in wealthy counties like Santa Clara from 2000 to 2013 could be explained at least in part by deliberate choices made by wealthy white and Asian parents, combined with wider access to better life options afforded to those with higher income and education. These choices may include prenatal and postnatal practices that prevent ASD from developing in the first place (Mumper 2013) or prevent birth complications that increase the risk of ASD (Jacobsen et al. 2017). They also may involve proactive early and intensive interventions leading to recovery and loss of ASD diagnosis, although the available evidence shows limited success for such childhood interventions, on the order of 3–25%, in achieving complete loss of diagnosis (Rogers et al. 2012; Fein et al. 2013; Camarata 2014). A related possibility is that early and intensive behavioral intervention may decrease the severity of the ASD diagnosis, even if it falls short of achieving complete loss of diagnosis (Dawson et al. 2010). In such cases, some higher income children may no longer qualify for services through DDS, which historically has served the more severe end of the spectrum.

An alternative explanation for the results in Figs. 2, 3, and 4 is that wealthy white and Asian parents are simply opting out of DDS in favor of privately funded services. California insurance law since October 2011 requires that every health care plan that provides hospital, medical, or surgical coverage shall also provide coverage for behavioral health treatment for autism, including applied behavior analysis (ABA) and other evidence-based behavior intervention programs (L&M Policy Research 2013). Medi-Cal (California’s Medicaid program) is exempt from this law. The law does not include specific age limits or dollar caps, but caps in other states are typically $36,000-$50,000 per year through ~ age 9 (L&M Policy Research 2013). It is therefore likely that wealthier families today obtain behavioral health treatment through private insurance, while lower income families rely on DDS. However, this private insurance mandate would only have affected the very youngest cohorts in our 2019 age-resolved snapshot, which spans birth years 1993–2013. Assuming a mean age of diagnosis of 3 years in the DDS system (Fountain and Bearman 2011), parents of children born in ~ 2009 onward would have been covered by the private insurance mandate. By similar logic, an earlier “mental health parity” insurance law passed in September 1999 would have applied to children born in ~ 1997 onward. However, while in principle that law required coverage of autism treatment, in practice it created confusion amongst insurers and ABA therapy continued to be funded primarily by the public sector, including schools and DDS regional centers (Lake et al. 2002). At the time of the current study, insufficient data were available to assess the extent to which opt-out of DDS by wealthy parents might contribute to the trends observed in our study, since DDS does not collect information about those who decline services for which they otherwise would be eligible (DDS, personal communication).

Some indication of the underlying cause of ASD can be found in common denominators among risk factors for autism as well as promising biomarkers for autism. Risk factors and biomarkers of ASD generally point strongly toward immune inflammation and oxidative stress (Goldani et al. 2014; Bilbo et al. 2015). These can be reduced by practices that include healthier dietary choices (Sears 2015), avoidance of environmental toxins (Mumper 2013; Thompson et al. 2015), and mitigation of chronic psychological stress (Brenner et al. 2015; Liu et al. 2017). Such health-oriented practices tend to be more widely embraced by or accessible to the wealthy than the poor (Pampel et al. 2010; Rehm et al. 2016). The precise cause of the decline in ASD prevalence in wealthy whites since 2000 is unknown and beyond the scope of this study. However, it seems likely that more than one behavior, choice or other factor is at play.

Our study provides a rare glimpse of trends in Asian ASD prevalence. Asians are a small minority in most U.S. states and thus have not been the main focus of other studies that resolve ASD trends by race (CDC 2018; Nevison and Zahorodny 2019). However, Asians are the third largest component of the school population in California and are represented in a large number of counties in the DDS ASD dataset (i.e., not de-identified due to small counts). In the wealthy counties of the San Francisco Bay Area, Asians show similar trends to whites, with ASD prevalence rates that flatten (but do not actually decline as in the case of whites) in the most recent birth years. In other regions, including the Central Valley counties and large metropolitan areas like Sacramento, Los Angeles and San Diego, Asian trends more closely follow Hispanic trends, with steep increases in recent years that cause Asian prevalence to catch up to or surpass white prevalence (Supplementary Fig. S5). Statewide, Asian ASD prevalence is second only to black prevalence and exceeds ASD prevalence among both whites and Hispanics (Fig. 1). Like whites, the rate of change in ASD prevalence among Asians, is significantly and inversely correlated to mean income at the county level over birth year 2000–2013, although unlike whites, absolute prevalence over this period is not correlated (Figs. 3, 4). Notably, the term Asian encompasses a wide range of cultures and ethnicities, encompassing both recent immigrants and long-time American citizens, and spanning from the Middle East to India to the Far East and the Pacific Islands. A breakdown of how these different groups are distributed in California at the county level may provide insight in future work into the disparate trends among Asians by county.

Some limitations of our study include the uncertainty in the denominators of NCES race and county specific prevalence, including the need to extrapolate these denominators to report year 2019 across a 21-year range of birth cohort years. However, the NCES populations are either flat or smoothly varying for most counties and races, such that the denominators provide a stable estimate of the true race and county specific populations, i.e., changes in the ASD counts rather than the NCES denominators are the main drivers of the prevalence trends. Another limitation is that the NCES denominators include only public school populations. As a result, NCES may underestimate the total population in counties with large private school populations. To the extent that private schools would be expected primarily in wealthy areas and that small denominators lead to the overestimate of ASD prevalence, the relationships with wealth may be even stronger than shown here.

Some limitations of the numerators (i.e., the ASD counts), are that DDS traditionally has covered the more severe end of the ASD spectrum. It is therefore possible that the relationships with wealth are most pronounced with respect to severe autism; the current study cannot assess whether milder ASD prevalence is also inversely correlated to county wealth. In addition, this is an ecological, population-based study without access to personally identifying information about each ASD case. Furthermore, the dataset is organized by current county residence, and provides no information about the county of birth. Finally, the DDS data do not include those who may have been enrolled prior to 2019 in the DDS caseload and subsequently dropped out.

A strength of the current study is its ability to separate by race and thereby untangle confounding influences in the total DDS dataset for all races. The relationship between mean ASD prevalence over birth year 2000–2013 and county income is substantially stronger, for example, among whites alone (p < 0.001) than among all races (p = 0.05) (Fig. 3). The relationship between the rate of change in prevalence and county income is also stronger for whites alone (p < 0.001) than for all races (p = 0.002) (Fig. 4). These distinctions occur largely because Hispanics comprise more than 50% of the California public school population and, unlike whites, their county-level ASD trends show neither a change point around birth year 2000 nor a significant relationship with county income (Figs. 3, 4). In addition, Hispanics have had relatively low ASD prevalence historically but recently have experienced a rapid increase (Nevison and Zahorodny 2019).

Current U.S. policy focuses on early diagnosis of ASD accompanied by supportive therapies, based on the premise that we do not know how to prevent or lower risk for ASD (CDC 2018). With ASD now affecting up to 3% of children (Zablotsky et al. 2017) and, in some states, nearly 5% of boys (CDC 2018), there is an urgent need to understand what wealthy California parents are doing or have access to that may be lowering their children’s risk, and what changes occurred around 2000 that may have affected these parents’ behavior or options. Conversely, there is an equally urgent need to understand the factors that may lead to increased risk of ASD among lower income populations, including factors that may be beyond their control, such as access to and autonomy over healthy food, health care, stressful environments and toxic exposures.

## Conclusion

In the wealthiest areas of California, including Santa Clara County in the Silicon Valley and the coastal counties extending from Monterey through San Francisco and north to Marin and Sonoma, white ASD prevalence trends show a striking change around birth year 2000. Following years of steady increases in prevalence in the 1990s, which are similar across all California counties, ASD prevalence among whites and Asians in these wealthy counties flattened and, for whites, actually decreased over birth year 2000–2013. In contrast, in lower income counties, particularly in the Central Valley and northern and eastern areas of California, white and Asian ASD prevalence increased at a similar or accelerating rate over birth years 2000–2013 compared to birth years 1993–2000. These patterns are not evident in the ASD trends among Hispanics, who comprise the majority of California’s school population. Rather, Hispanic ASD prevalence increased steadily across 1993–2013. However, its absolute value generally remained lower than that of whites across the entire period, except in the wealthiest counties, where it surpassed white prevalence. ASD prevalence among blacks is more difficult to assess at the county level due to DDS privacy policies, since blacks are a small minority in most California counties. However, statewide black ASD prevalence is increasing rapidly and, at 1.8% in birth year 2013, was the highest of all race/ethnicity groups. Overall, the results suggest that the most wealthy and educated parents, starting around birth year 2000, began either to opt out of DDS in favor of private services and/or to make choices and access options that lowered their children’s likelihood of being diagnosed with the severe forms of ASD historically served by DDS.

## Electronic supplementary material

Below is the link to the electronic supplementary material.

Supplementary file1 (DOCX 2939 kb)

Supplementary file2 (XLSX 44 kb)

Supplementary file3 (XLSX 37 kb)
