# Supplementary material for: California Autism Prevalence by County and Race/Ethnicity: Declining Trends Among Wealthy Whites
Source: J Autism Dev Disord. 2020 Mar 19;50(11):4011–21. doi: 10.1007/s10803-020-04460-0 (PMC7557477; doi:10.1007/s10803-020-04460-0)

**­California autism prevalence by county and race/ethnicity: Declining trends among wealthy whites**

**SUPPLEMENTARY INFORMATION**

Supplementary File S1 presents plots of NCES denominators (Figures S1 and S2) and DDS/NCES ASD prevalence among whites, Hispanics and all races for 36 California counties (Figures S3). This file also presents plots of black and Asian ASD prevalence, each compared to white prevalence, in selected counties with available data (Figures S4 and S5). Finally, plots of race and county specific mean ASD prevalence and rate of change in prevalence vs. county income are shown for both birth year periods, 1993-2000 and 2000-2013 (Figure S6 and S7).

Supplementary File S2 presents NCES public school populations for 36 California counties projected to report year 2019, partitioned into 5 race/ethnicity groups: all races, whites, blacks, Asians and Hispanics for each birth cohort from 1993-2013. Supplementary File S3 provides estimated DDS/NCES ASD prevalence for the 2019 age-resolved snapshot, partitioned into 5 race/ethnicity groups and 36 California counties plus 10 additional county groups.

**Supplementary File1: Figures S1-S6**

Overview of Figures:

Figures S1 and S2 illustrate the estimation of race-resolved NCES total school population denominators, available through report year 2017 and extrapolated to report year 2019.

Figures S3-S5 show the complete set of ASD vs. birth year plots for all California counties and county groups. Fig S3 shows all races, whites and Hispanics. Figure S4 shows blacks and whites. Figure S5 shows Asians and whites.

Figure S6 shows mean ASD vs. mean county income for 4 race/ethnicity groups over each of two birth year intervals: 1993-2000 and 2000-2013.

Figure S7 shows rate of change in ASD vs. mean county income for 4 race/ethnicity groups over each of two birth year intervals: 1993-2000 and 2000-2013.

**Supplementary Figure S1** (pp. 2-3 below). NCES school populations for 36 California counties, distinguished by race/ethnicity and illustrating the extrapolation of the school populations for a selected birth cohort (2008) to report year 2019 (large triangles). The extrapolation to 2019 is necessary because the NCES data, which are used as denominators in the ASD prevalence calculation, are available only through the NCES report from Fall 2017, while the DDS ASD numerators are from Fall 2019.


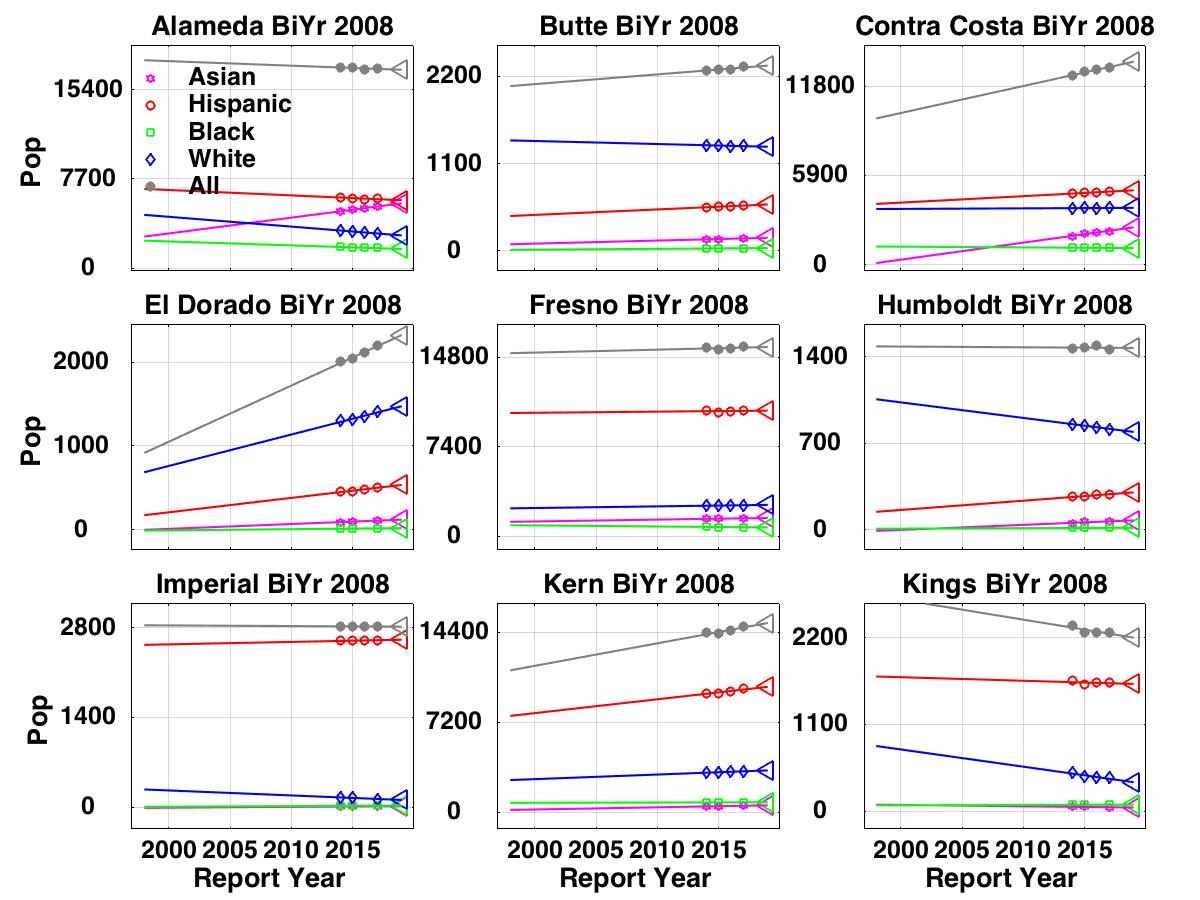


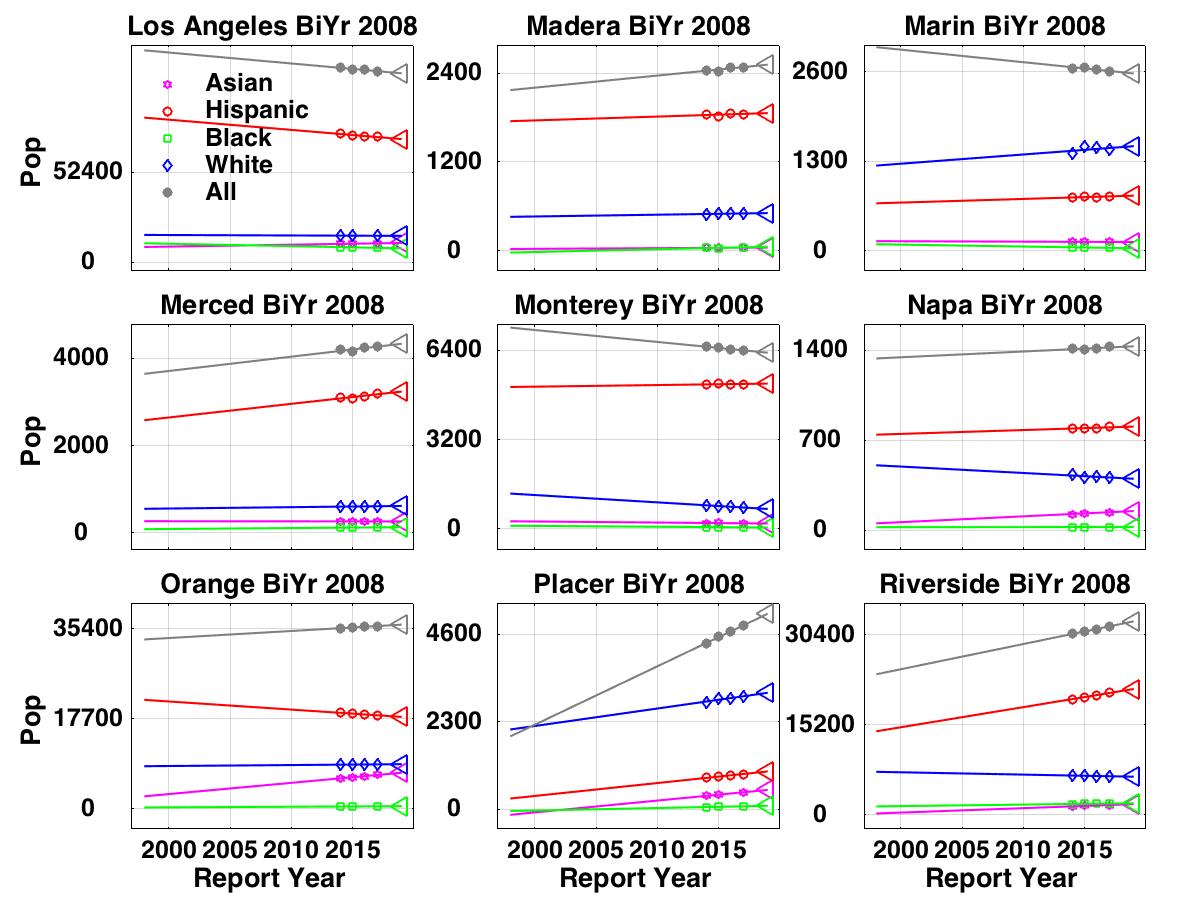


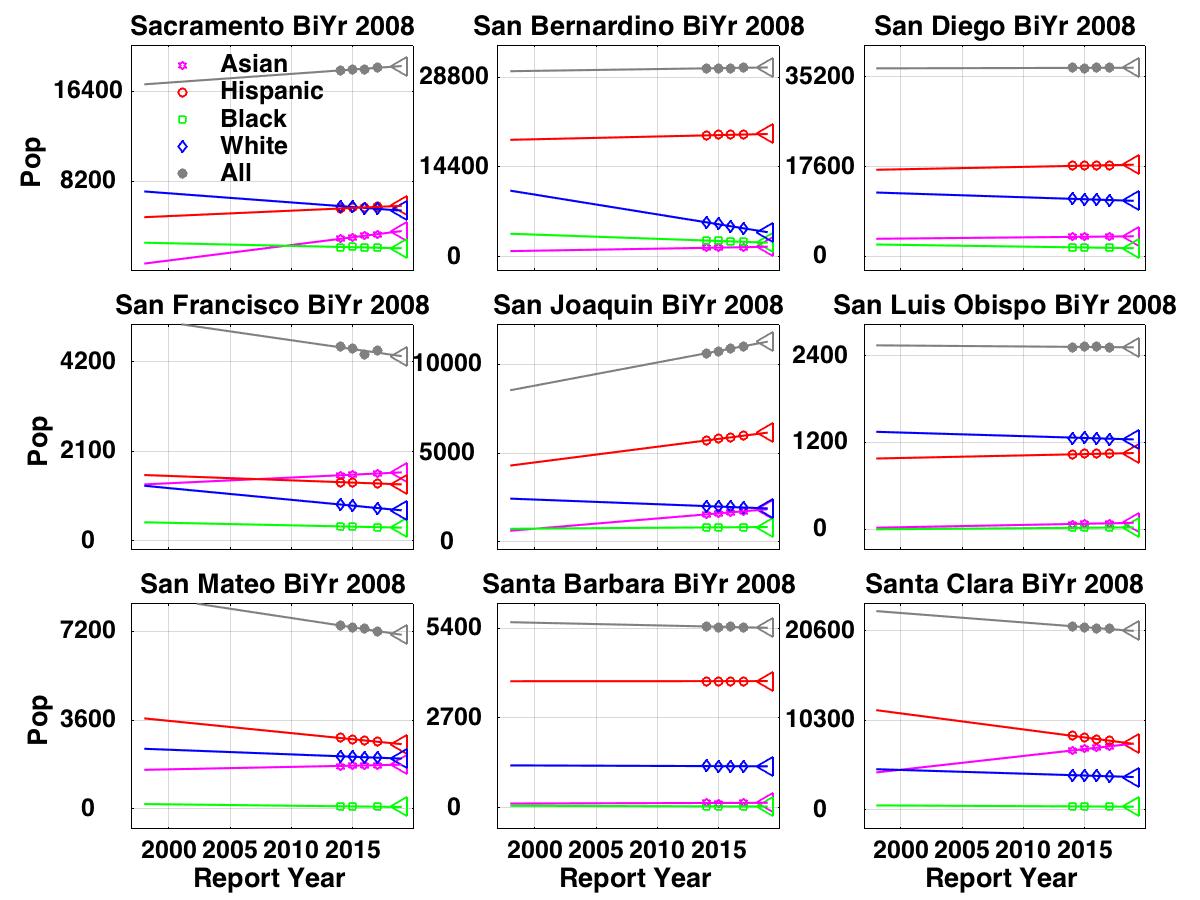


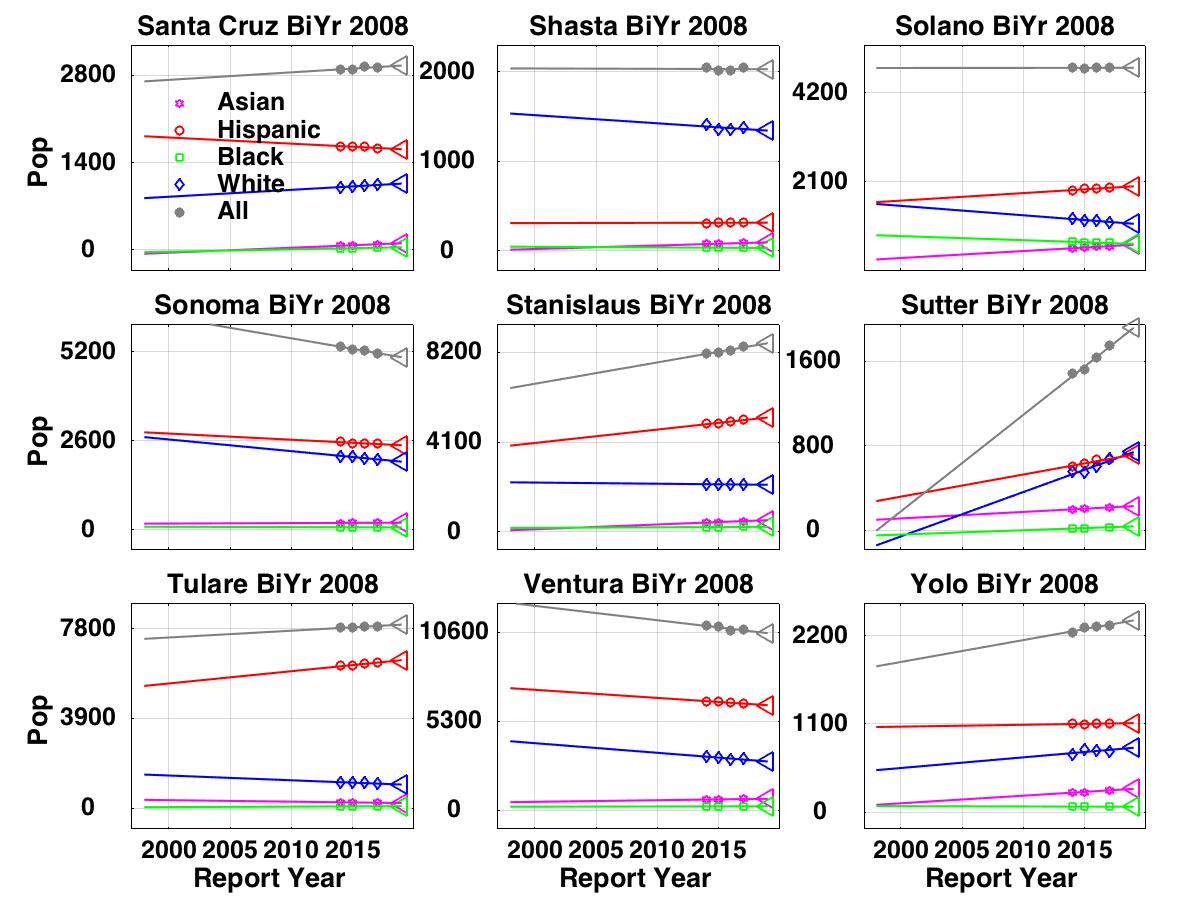


Figure S2. Same as Figures S1 except showing NCES school populations statewide and for a selected California county (Santa Clara), distinghished by race/ethnicity and illustrating the extrapolation of the school population to 2019 for selected birth cohorts.


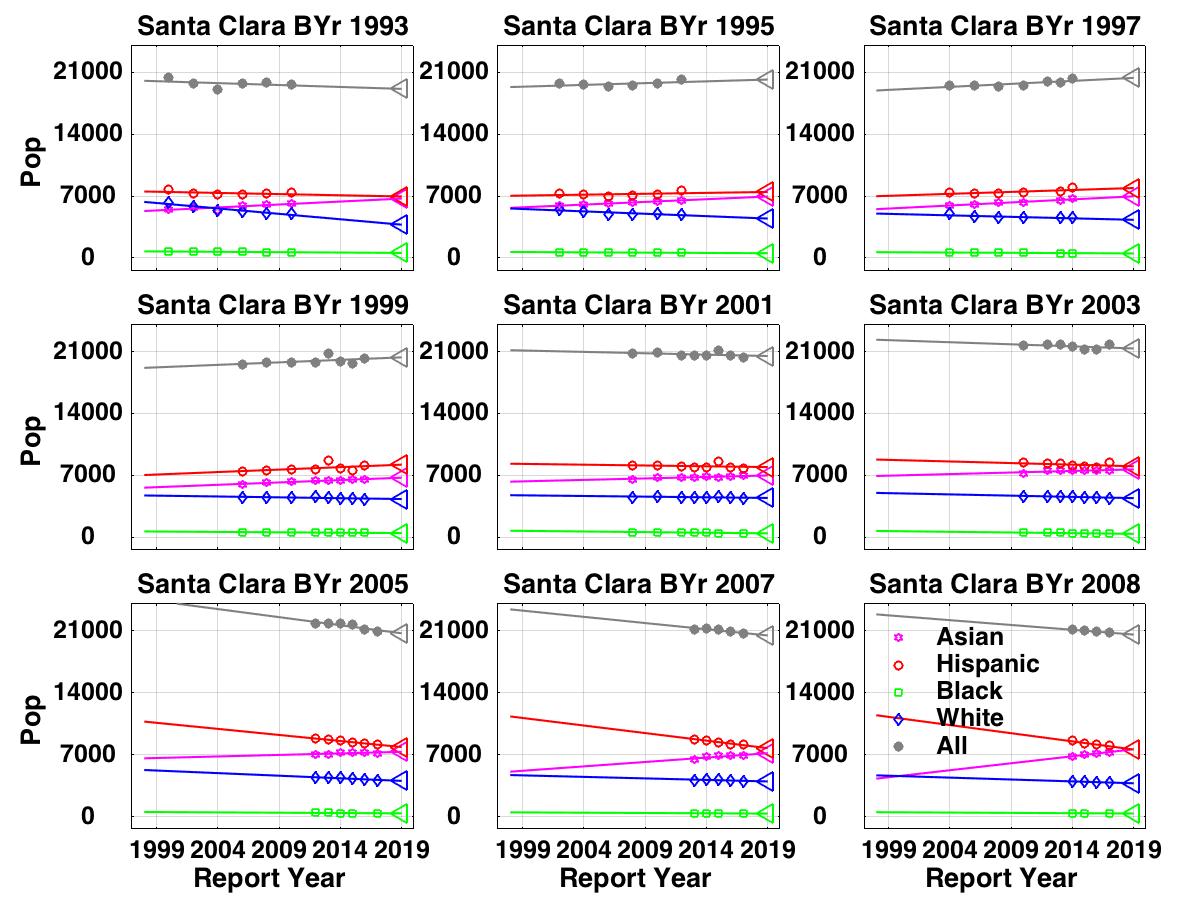


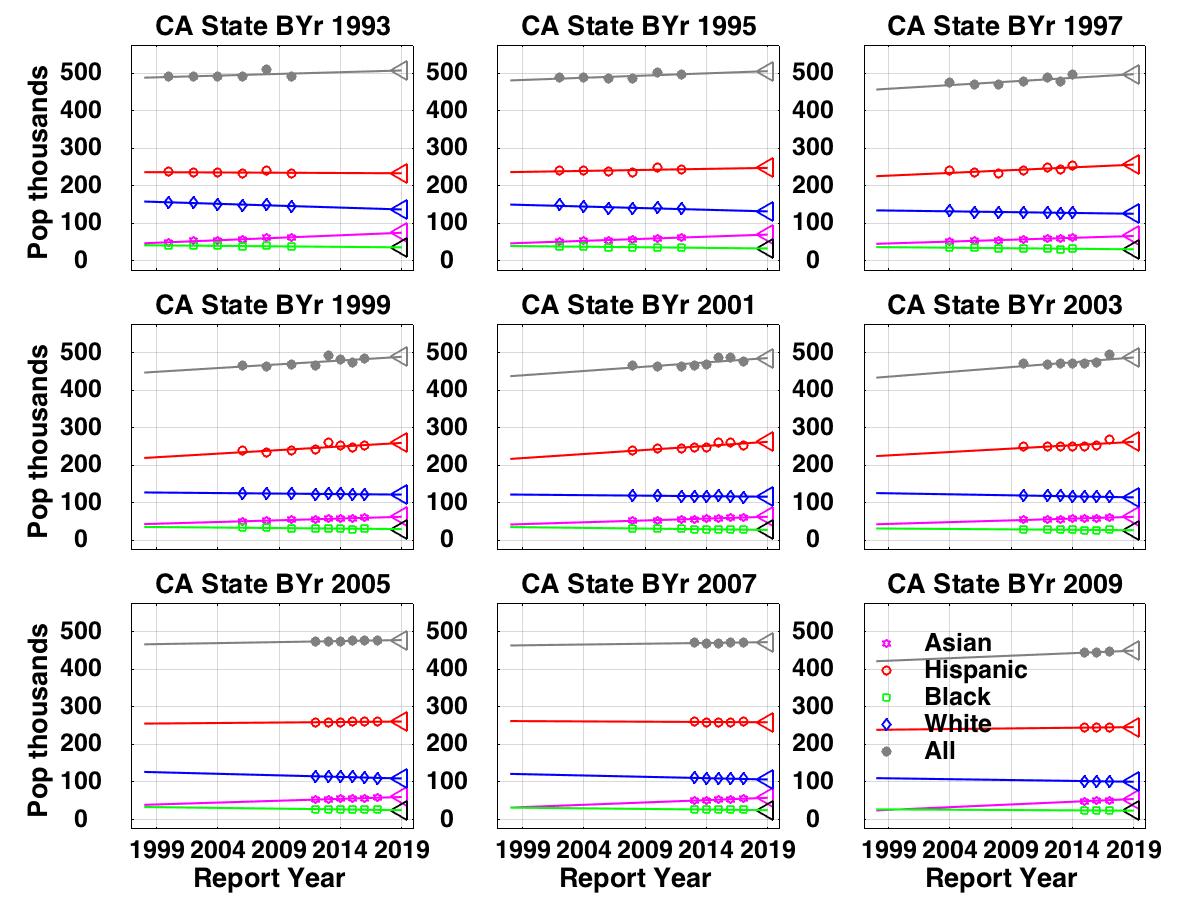


**Figure S3.** ASD prevalence vs. birth year for all races (gray crosses), Hispanics (red circles) and whites (blue diamonds) in 45 California counties or county groups (defined below). Linear regressions distinguish time trends for 2 separate periods: 1993-2000 (dashed) and 2000-2013 (solid). Definition of county groups:

East/South Bay = Alameda and Santa Clara

Sierra Foothills = Butte, El Dorado, Placer and Sutter

South Central Valley = Fresno, Kern, Kings, Madera and Tulare

Monterey to SF Coast = Monterey, Santa Cruz, San Mateo and San Francisco

North San Joaquin Valley = Merced, San Joaquin and Stanislaus

Delta Northeast = Napa, Solano and Yolo

South Central Coast = Santa Barbara and San Luis Obispo


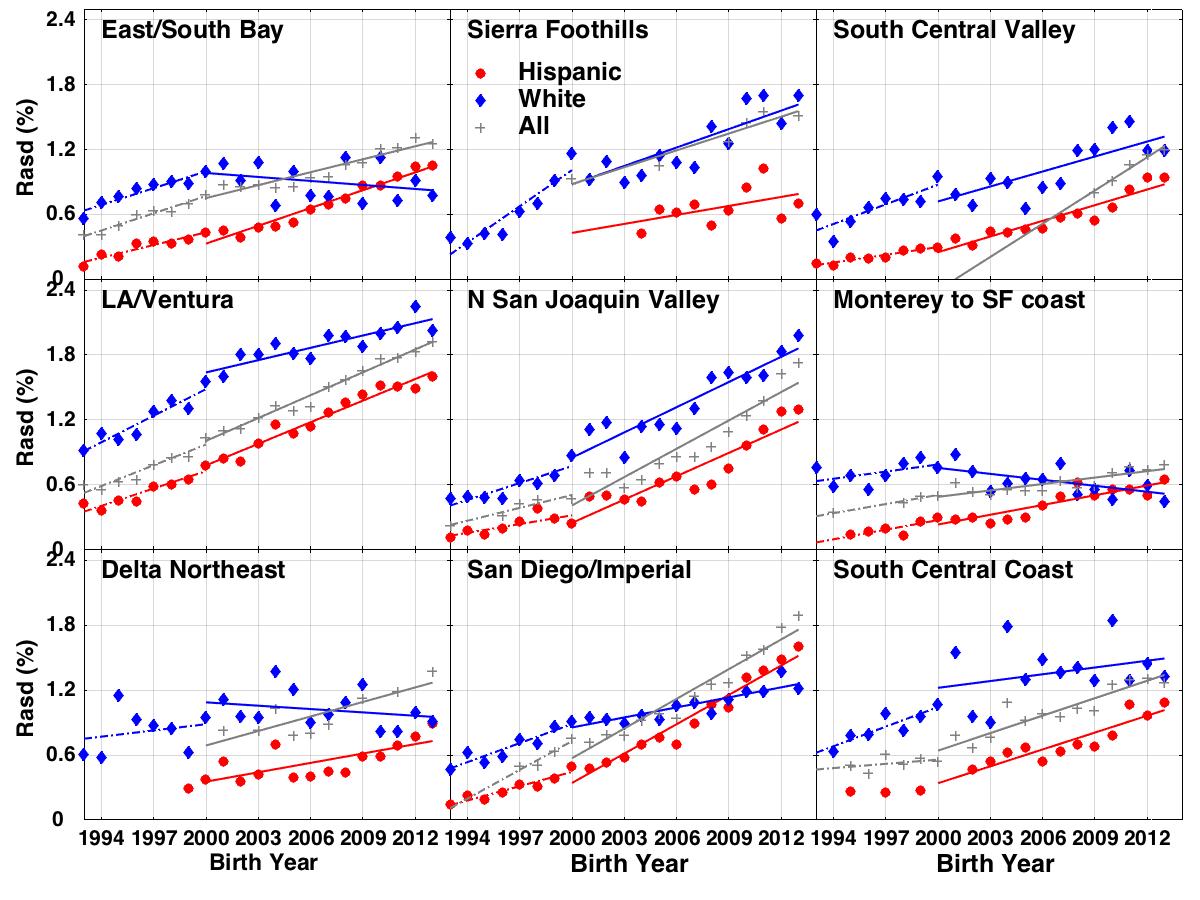


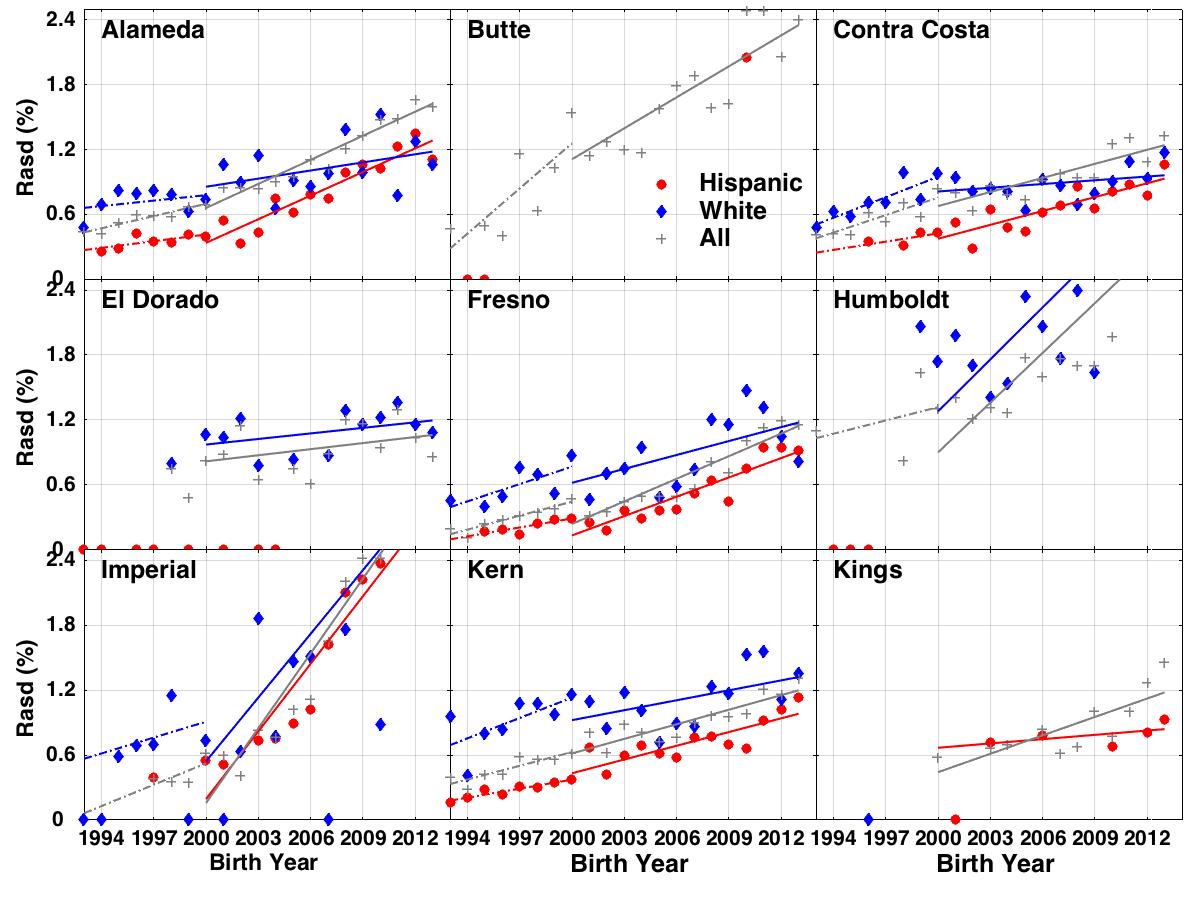


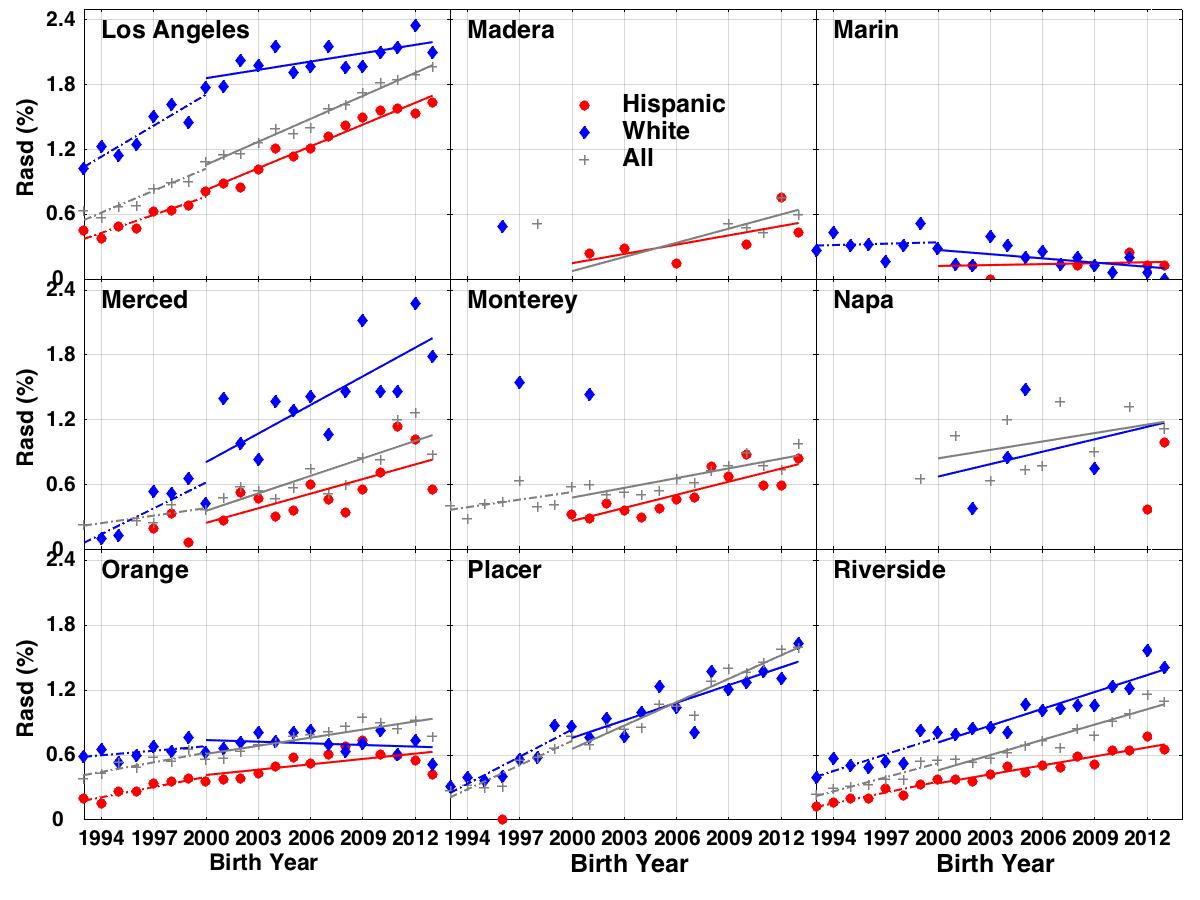


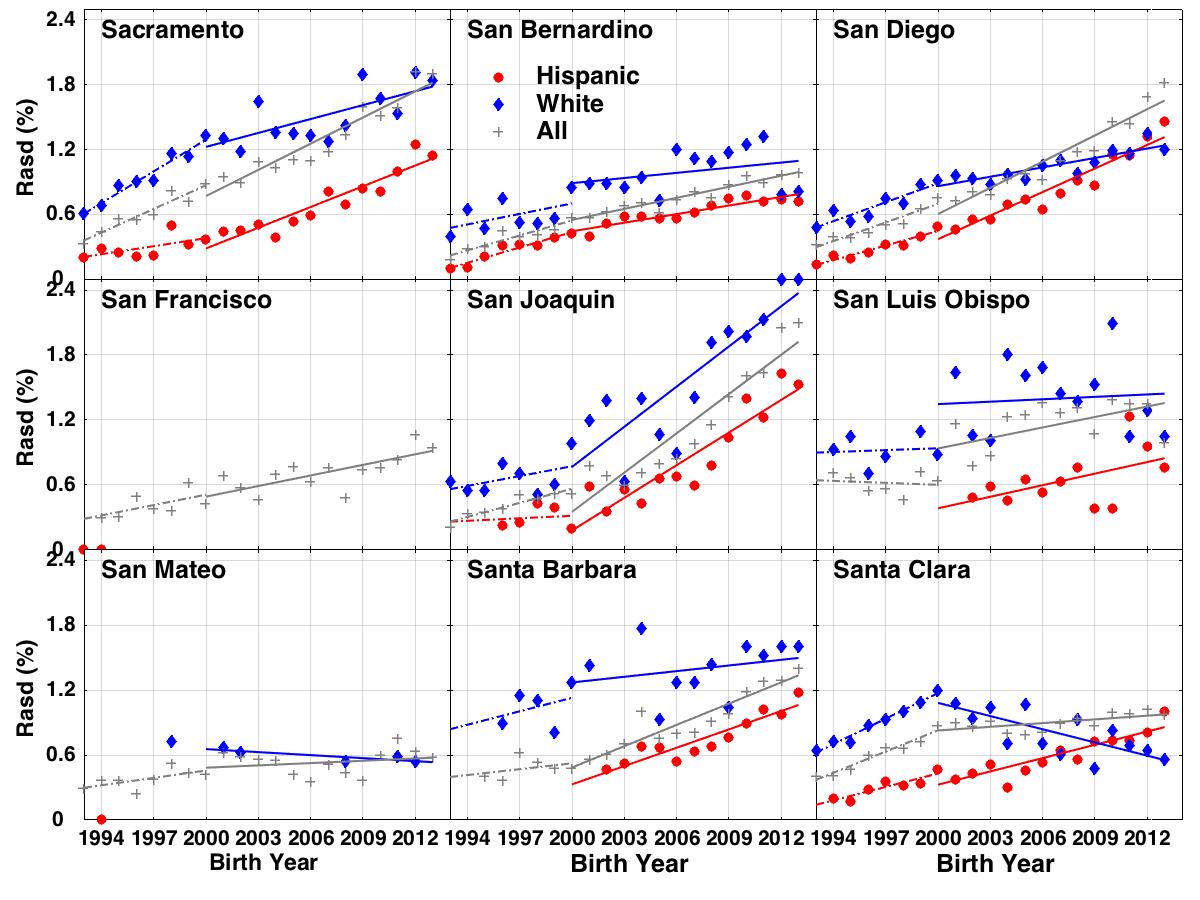

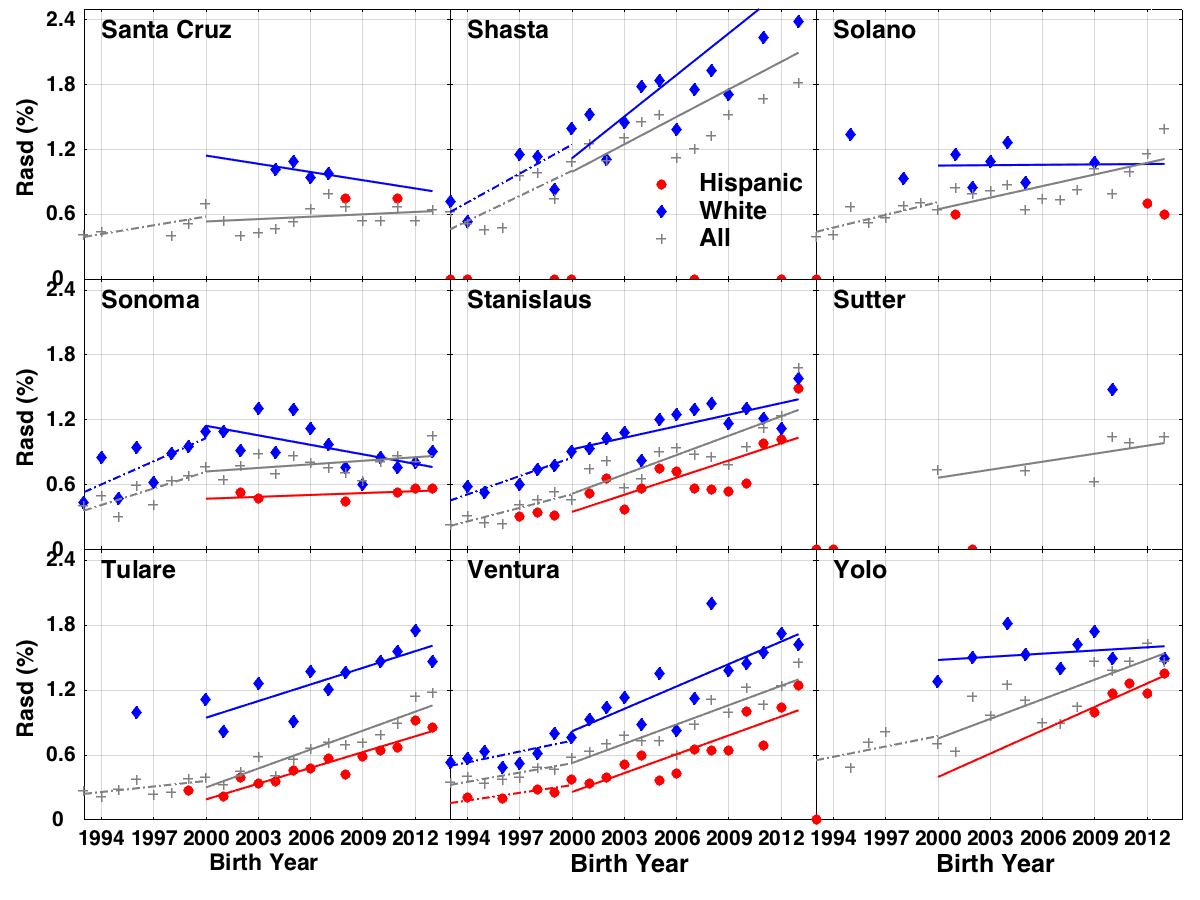


**Figure S4.** ASD prevalence vs. birth year for blacks (green squares) and whites (blue diamonds) in 9 California counties or county groups (defined below) in which ASD data for blacks are available. Linear regressions distinguish time trends for 2 separate periods: 1993-2000 (dashed) and 2000-2013 (solid).

**
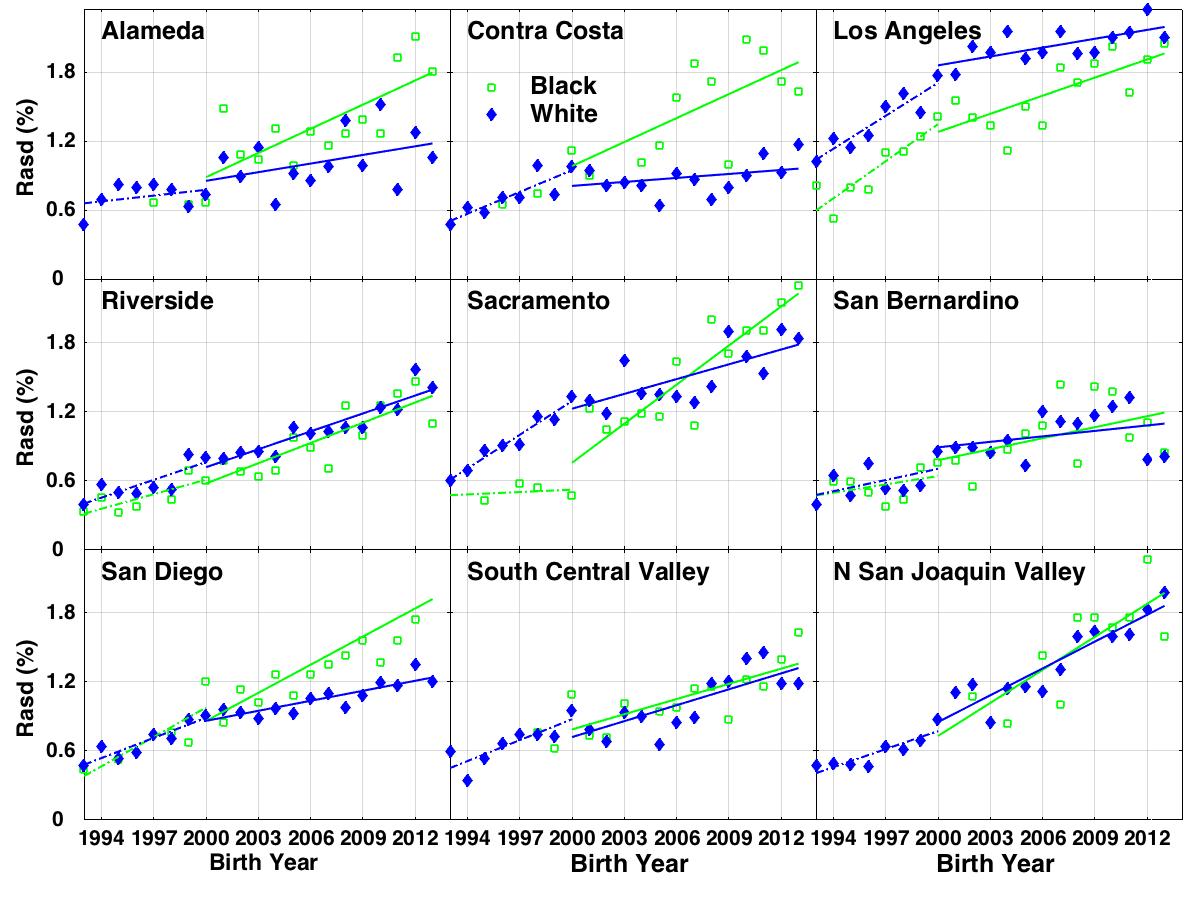
**

**Figure S5** (next page). Same as Figure 1 in main text, but for Asians (magenta stars) and whites (blue diamonds). (Note: Asian data are not available for Marin/Sonoma.) Also shown are 9 additional California counties or county groups in which ASD data for Asians are available.


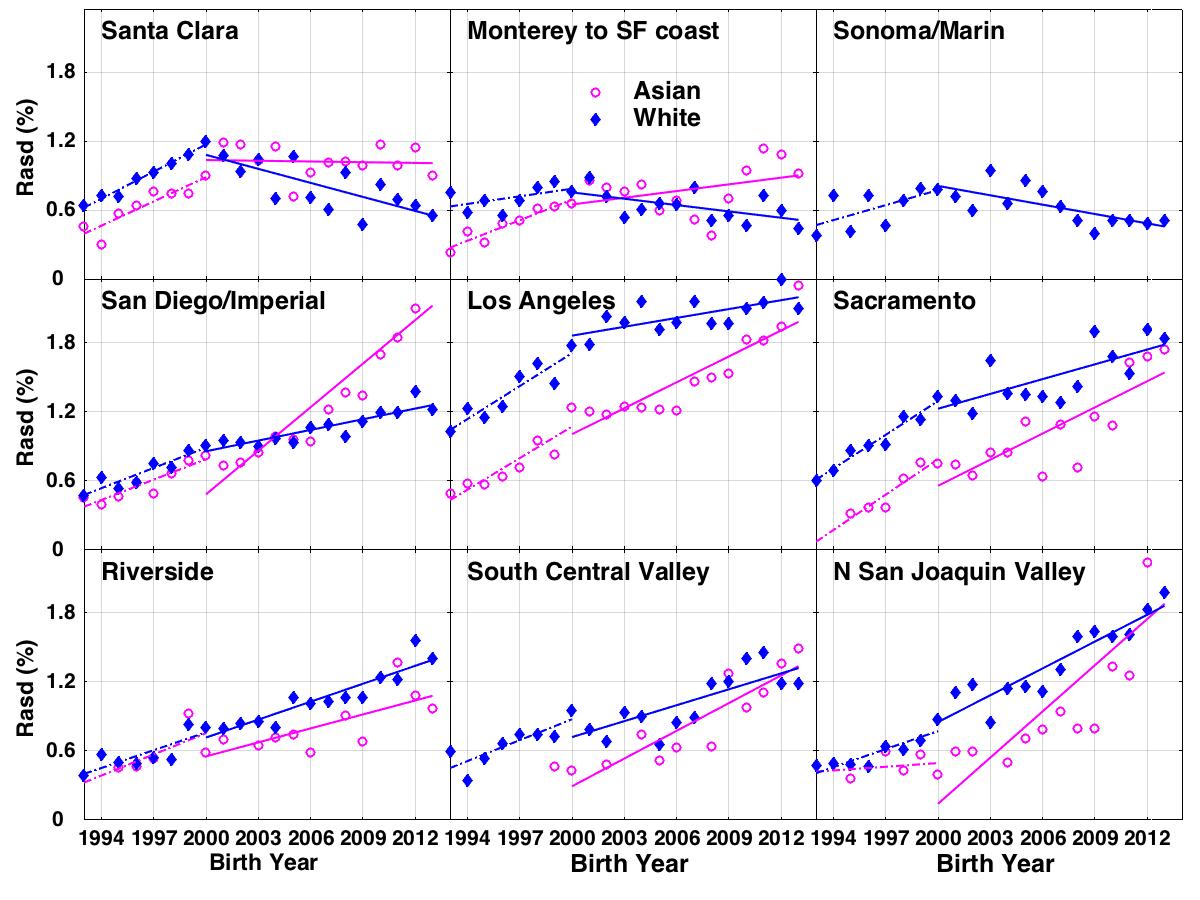

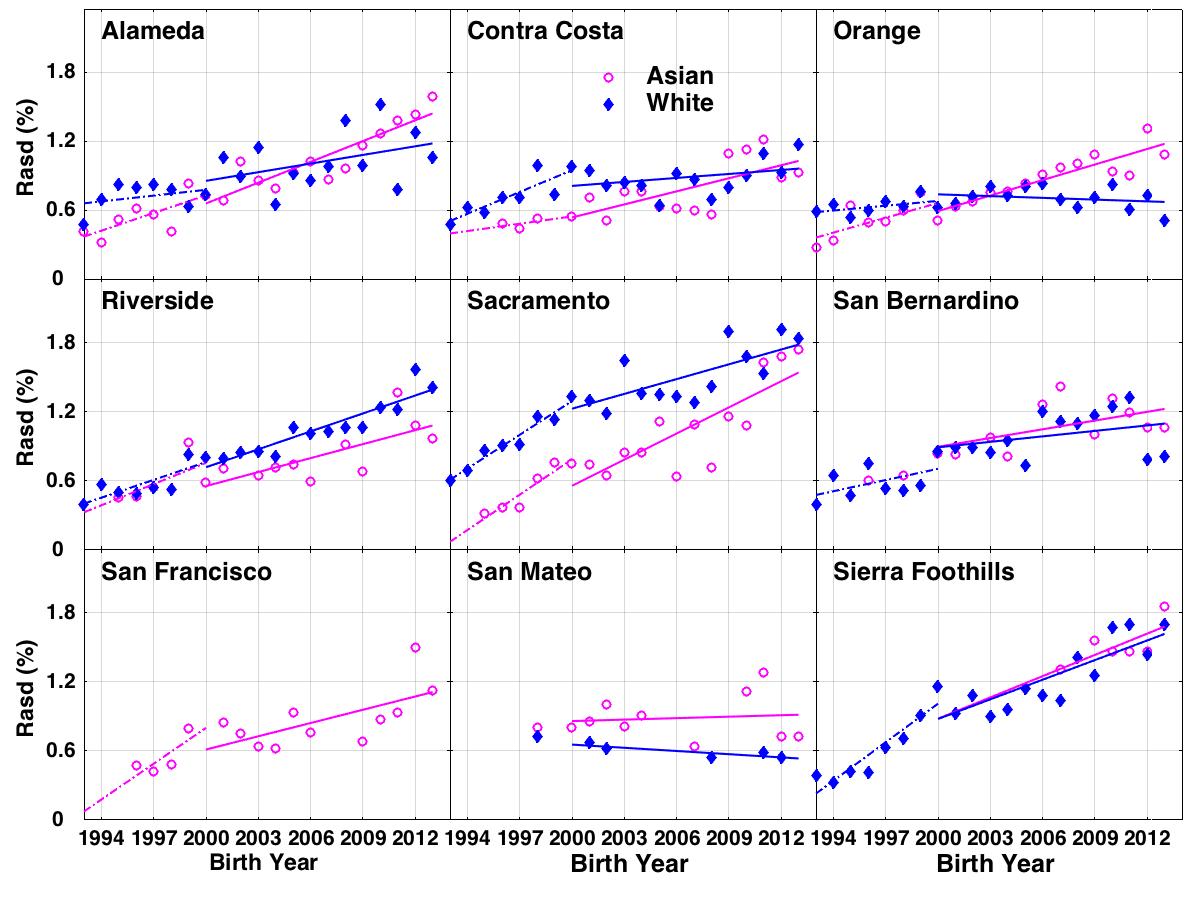


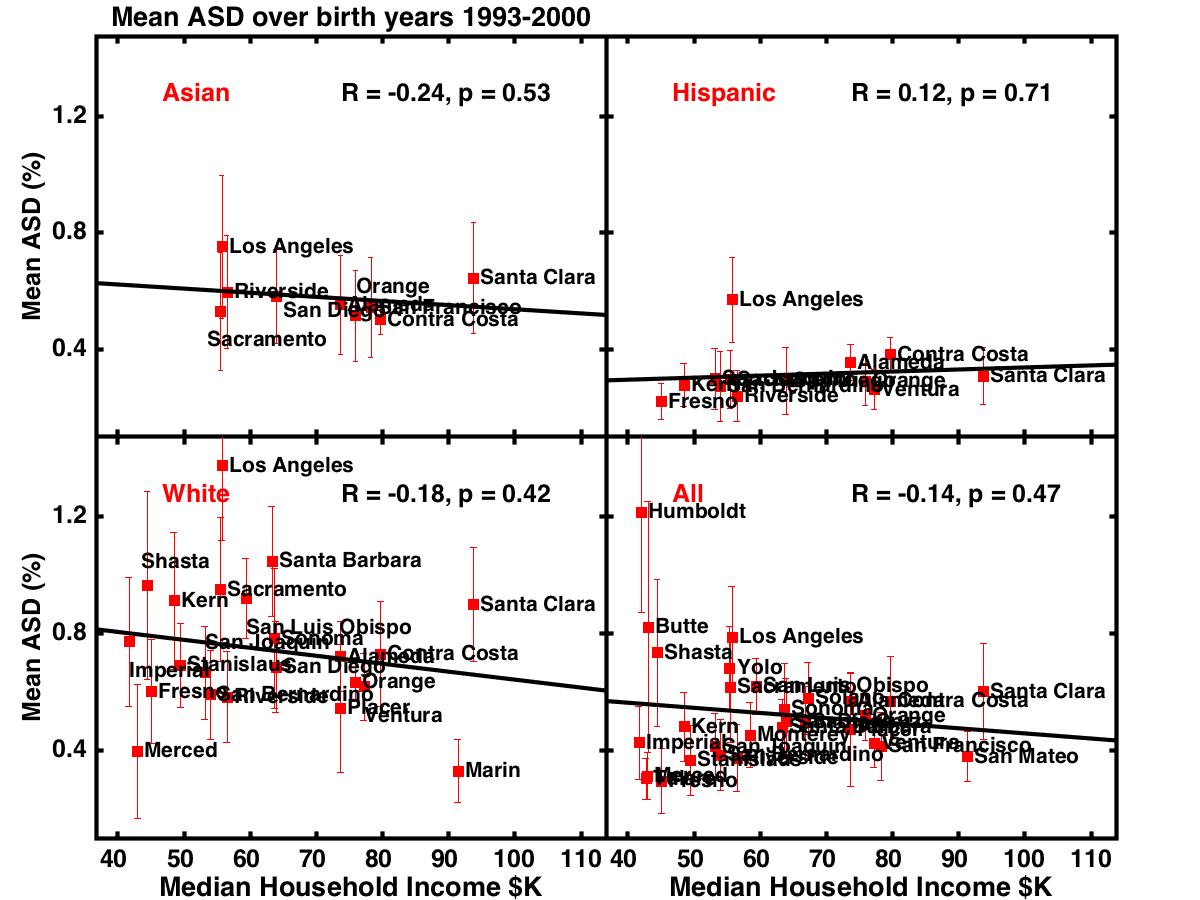


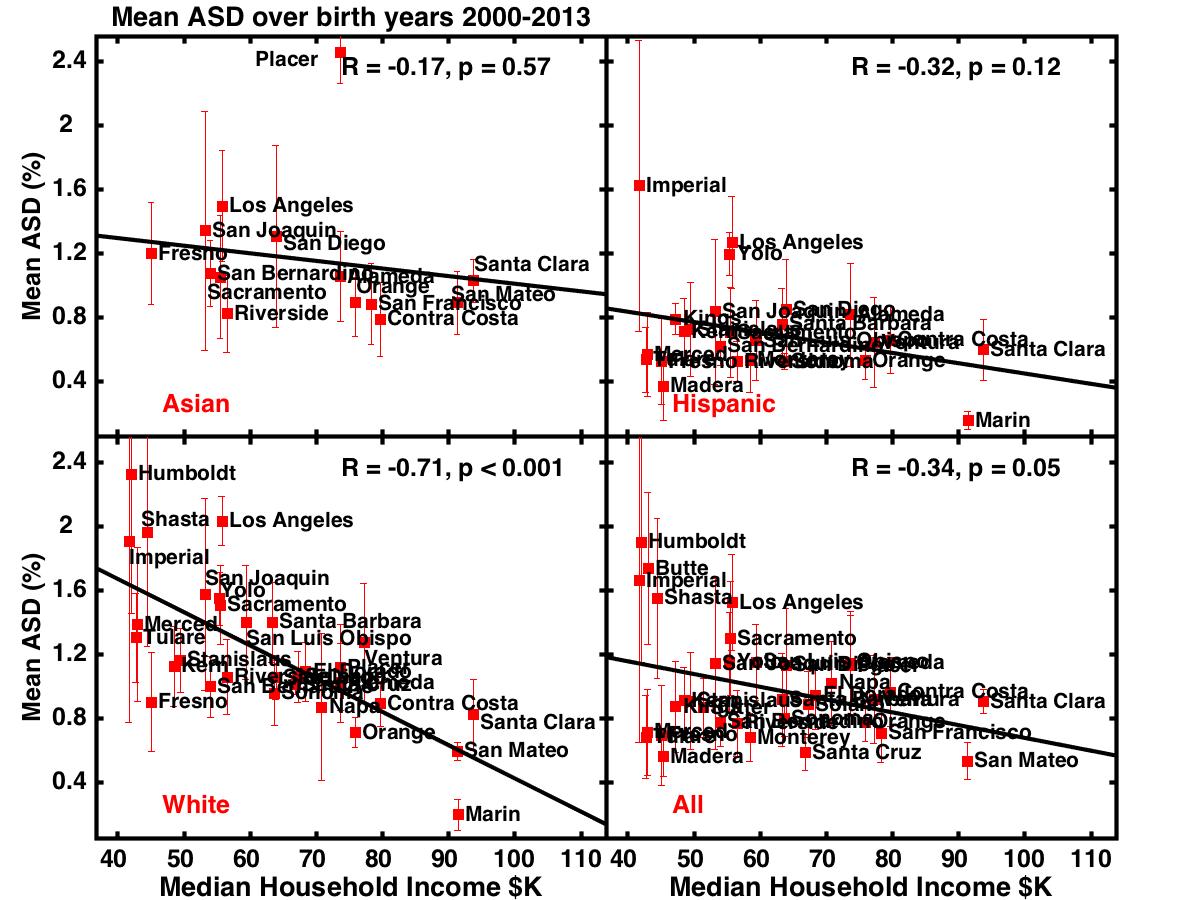


**Figure S6** (previous page). Mean ASD prevalence over birth years 1993-2000 (upper 4 panels) and birth years 2000-2013(lower 4 panels) by California county. Error bars show the standard deviation. (Note that the lower 4 panels repeat Figure 3, in order to illustrate the contrast between the two time periods.)

**Figure S7** (next page). Rate of change in ASD prevalence over birth years 1993-2000 (upper 4 panels) and birth years 2000-2013 (lower 4 panels) by California county, estimated using linear regression. Error bars show the error in the linear regression slope. Counties which the slope error is < 40% of the slope. Results are shown for 4 different race/ethnicity groups. (Note that the lower 4 panels repeat Figure 4, in order to illustrate the contrast between the two time periods.)


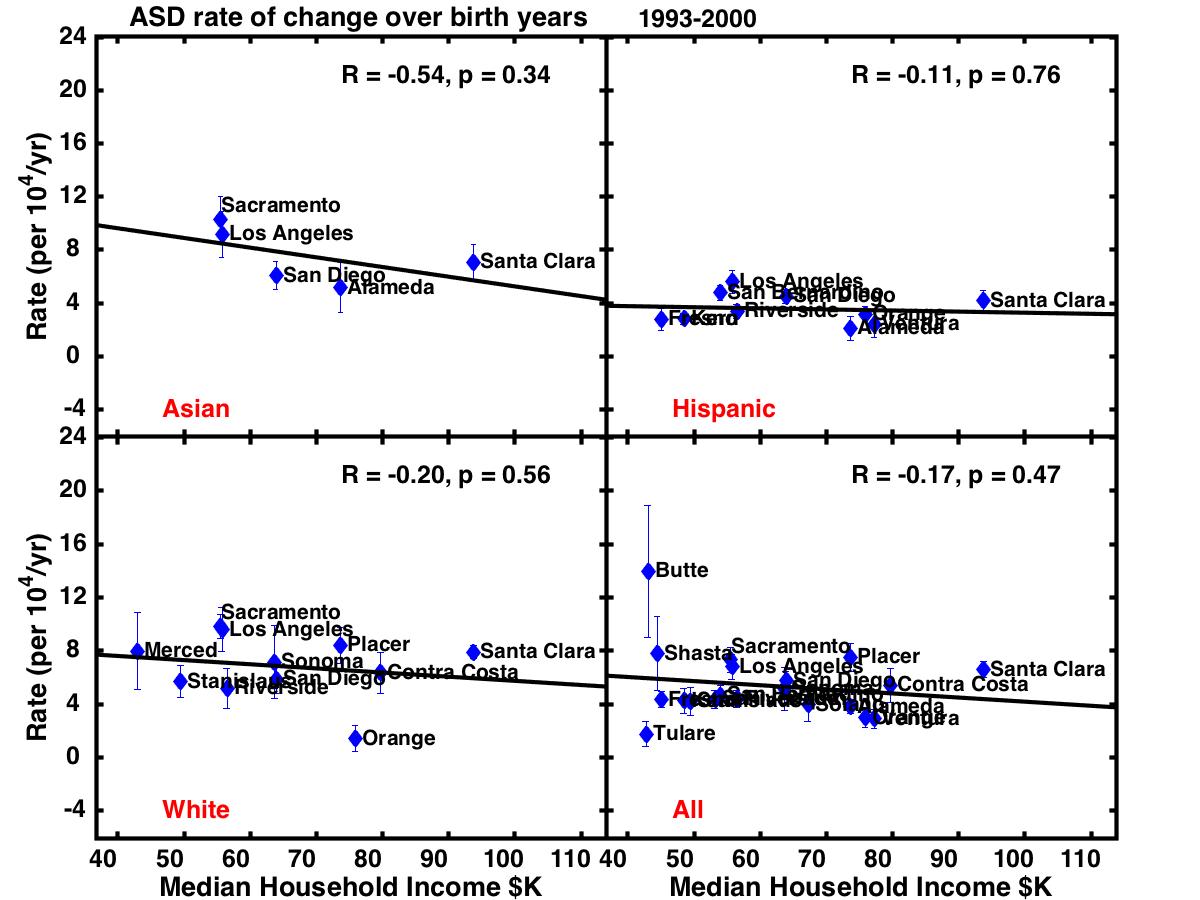


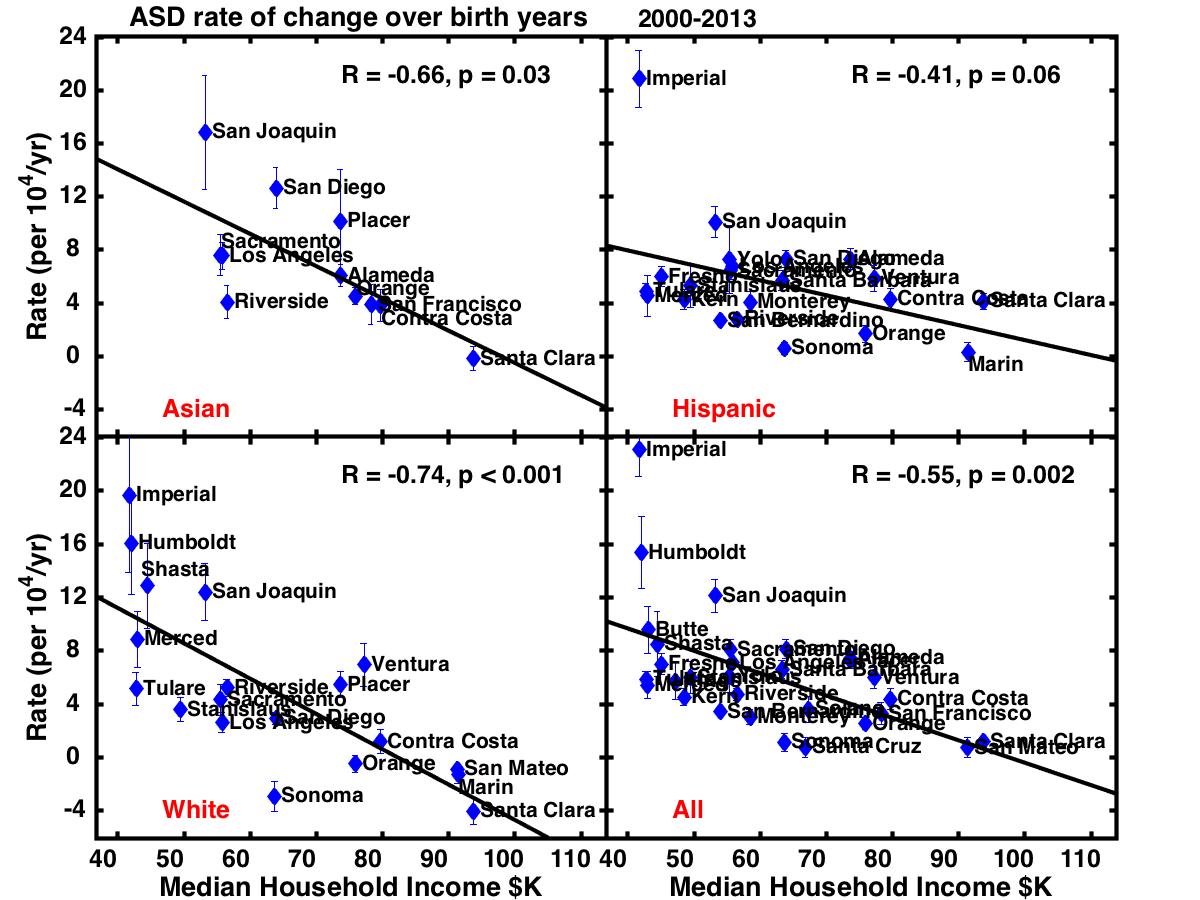

Supplement: Supplementary file 1 — Supplementary file1 (DOCX 2939 kb) [file 10803_2020_4460_MOESM1_ESM.docx]
